# OTUB1 inhibits the ubiquitination and degradation of FOXM1 in breast cancer and epirubicin resistance

**DOI:** 10.1038/onc.2015.208

**Published:** 2015-07-06

**Authors:** U Karunarathna, M Kongsema, S Zona, C Gong, E Cabrera, A R Gomes, E P S Man, P Khongkow, J W-H Tsang, U-S Khoo, R H Medema, R Freire, E W-F Lam

**Affiliations:** 1 Department of Surgery and Cancer, Imperial College London, Hammersmith Hospital Campus, London, UK; 2 Department of Pathology, Li Ka Shing Faculty of Medicine, The University of Hong Kong, Hong Kong SAR, China; 3 Unidad de Investigación, Hospital Universitario de Canarias, Instituto de Tecnologías Biomédicas, Ofra s/n, La Laguna, Tenerife, Spain; 4 Department of Clinical Oncology, Li Ka Shing Faculty of Medicine, The University of Hong Kong, Hong Kong SAR, China; 5 Division of Cell Biology, The Netherlands Cancer Institute, Amsterdam, The Netherlands

## Abstract

The forkhead transcription factor FOXM1 has a key role in DNA damage response, and its deregulated overexpression is associated with genotoxic drug resistance in breast cancer. However, little is known about the posttranslational mechanisms by which FOXM1 expression is regulated by genotoxic agents and how they are deregulated in resistant cells. Initial co-immunoprecipitation studies verified previous proteomic analysis finding that the OTUB1 is a novel FOXM1-interacting protein. Western blot analysis showed that both OTUB1 and FOXM1 expression reduced upon genotoxic agent treatment in MCF-7 cells, but remained relatively constant in resistant cells. FOXM1 expression reduced upon OTUB1 depletion by siRNA and increased with OTUB1 overexpression in MCF-7 cells, arguing that OTUB1 positively regulates FOXM1 expression. In agreement, co-immunoprecipitation experiments demonstrated that FOXM1 expression is associated with OTUB1 binding but inversely correlates with conjugation to the protein degradation-associated Lys-48-linked ubiquitin-chains. Overexpression of wild-type (WT) OTUB1, but not the OTUB1(C91S) mutant, disrupted the formation of Lys48-linked ubiquitin-conjugates on FOXM1. Importantly, knockdown of OTUB1 by siRNA resulted in an increase in turnover of FOXM1 in MCF-7 cells treated with the protein synthesis inhibitor cycloheximide, whereas overexpression of WT OTUB1, but not the OTUB1(C91S) mutant, significantly enhances the half-life of FOXM1. In addition, proliferative and clonogenic assays also show that OTUB1 can enhance the proliferative rate and epirubicin resistance through targeting FOXM1, as OTUB1 has little effect on FOXM1-deficient cells. The physiological relevance of the regulation of FOXM1 by OTUB1 is further underscored by the significant correlations between FOXM1 and OTUB1 expression in breast cancer patient samples. Cox-regression survival analysis indicates that OTUB1 overexpression is linked to poorer outcome in particular in patients treated with chemotherapy. Collectively, these data suggest that OTUB1 limits the ubiquitination and degradation of FOXM1 in breast cancer and has a key role in genotoxic agent resistance.

## Introduction

Breast cancer is one of the most prevalent causes of death in women worldwide. Genotoxic anti-cancer agents, including anthracyclines, platinum compounds, methylating agents and ionizing irradiation, are used widely to treat breast cancer patients who are not suitable for hormonal therapy and those with advanced or metastatic cancer. These genotoxic agents are also often used in the adjuvant setting, particularly after surgery, to prevent the return of the disease. However, resistance to these agents often emerges in patients, and this leads to suboptimal efficacy and disease relapse.^1^ The cellular response to DNA damage is a key determinant of the efficacy of these genotoxic agents, and these reactions include initiation of DNA damage repair response, cell cycle-checkpoint activation and induction of apoptosis or senescence. These processes ultimately govern cell fate and sensitivity to radiotherapy or chemotherapy. Conversely, a deregulated DNA damage response can lead to resistance to these anticancer agents.

Substantial evidence has accumulated to indicate that the Forkhead box M1 (FOXM1) transcription factor has a central role in cell proliferation, migration, invasion, angiogenesis, stem cell renewal, DNA damage repair and cellular senescence, which impact tumour initiation, progression, metastasis, angiogenesis and drug sensitivity. Recent research also indicates that deregulated FOXM1 overexpression confers genotoxic and other chemotherapeutic agent resistance in cancer.^2, 3, 4, 5, 6, 7^ There is already good evidence that FOXM1 acts as a mediator of DNA damage response as well as a modulator of genotoxic agent sensitivity.^4, 5, 6, 8, 9, 10^ Even though deregulated FOXM1 overexpression is considered key to the development of genotoxic agent resistance, the specific mechanisms involved in FOXM1 deregulation remain unknown. Therefore, a better understanding of the mechanisms that regulates FOXM1 expression in response to genotoxic agents and how FOXM1 is deregulated in resistant cancer cells is of importance for designing new therapeutic approaches directed to this degradation pathway.

Epirubicin is an anthracycline genotoxic drug commonly used for treating breast cancer.^11^ FOXM1 is downregulated by epirubicin at the transcriptional levels in breast cancer cells.^8, 12, 13^ However, the fact that FOXM1 protein expression declines at a faster kinetics when compared with its mRNA transcripts in response to genotoxic agents indicates that posttranscriptional mechanisms have a central role in regulating its DNA-damaging agent response in breast cancer cells.^8, 12^ In agreement, we have also shown recently that upon epirubicin treatment, FOXM1 is modified through SUMOylation, which leads to its ubiquitination and degradation through the ubiquitin-proteasome proteolytic pathway.^10^

Ubiquitination is a posttranslational modification that confers a range of protein regulatory functions, including targeting a substrate protein for degradation, modifying its activity, adjusting its function, changing its subcellular location and altering protein–protein interactions. Ubiquitination is a dynamic process and can be reversed by deubiquitination, which is catalysed by a family of proteins that are ubiquitin hydrolases or deubiquitinating enzymes called DUBs. OTUB1 (OTU domain-containing ubiquitin aldehyde-binding proteins 1; also called Otubain 1) belongs to the ovarian tumour domain protease (OTU) subfamily of DUBs. OTUB1 can negatively regulate ubiquitination and control protein stability and activity via a non-canonical mechanism as well as the conventional catalytic process. More specifically, OTUB1 can function as a cysteine protease that hydrolyses the isopeptide bond between ubiquitin and the target protein or another ubiquitin molecule. Alternatively, OTUB1 can also operate through a non-canonical mechanism independently of its catalytic activity, by inhibiting the activity of E2 ubiquitin-conjugating enzymes.

To explore the mechanisms by which FOXM1 expression is modulated at the posttranslational level as well as the molecules involved, we performed mass spectrometry analysis of protein complexes co-immunoprecipitated with FOXM1 in the breast carcinoma MCF-7 cell line and identified the deubiquitinase OTUB1 as a novel FOXM1-interacting factor. Given our previous finding that genotoxic agents cause FOXM1 SUMOylation, ubiquitination and degradation, these observations led us to hypothesize that OTUB1 promotes FOXM1 expression at the posttranslational level to potentiate genotoxic agent resistance. In this study, we examined the potential role of OTUB1 in the regulation of FOXM1 expression in genotoxic drug-sensitive and -resistant breast cancer cells.

## Results

### OTUB1 interacts with FOXM1 in breast cancer cells

**Figure 1:**
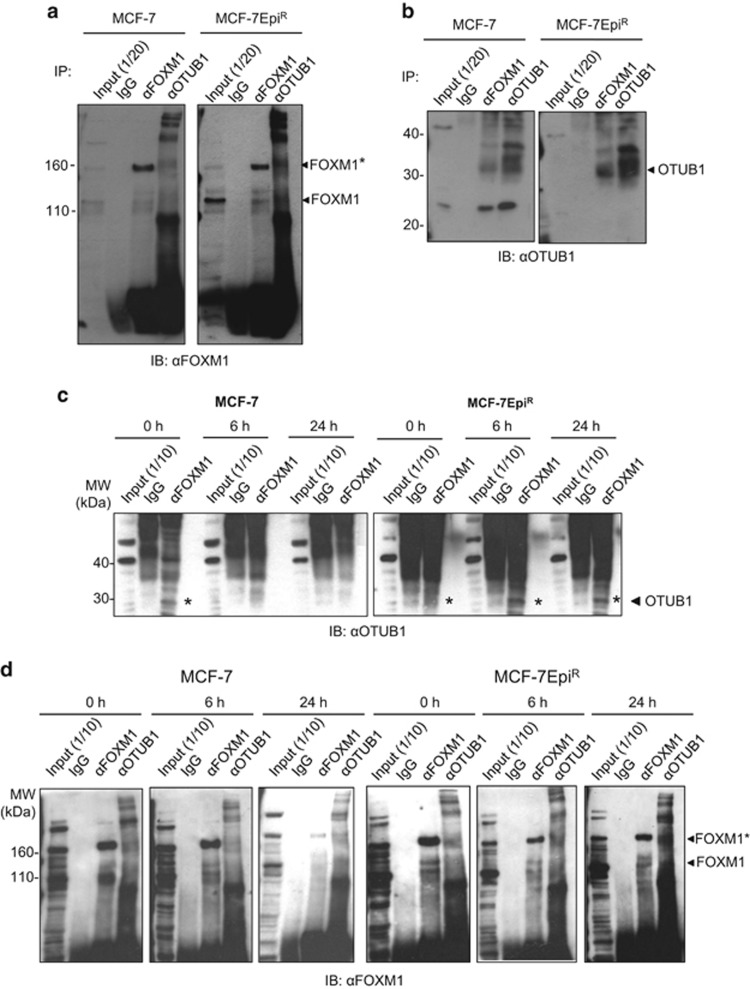
FOXM1 complexes with OTUB1 in MCF-7 and MCF-7Epi^R^ cells. Co-immunoprecipitation (co-IP) was performed with an IgG antibody control, a FOXM1 (α or anti-FOXM1) or an OTUB1 (αOTUB1) antibody on lysates from MCF-7 and MCF-7Epi^R^ cells; Inputs (1/20 of IP), and IP products with IgG and specific antibodies were resolved on western blot and probed for (**a**) FOXM1 and (**b**) OTUB1. FOXM1* represents a FOXM1 species associated with its SUMOylation. (**c**) MCF-7 and MCF-7Epi^R^ cells were treated with epirubicin (1 μM) for 0, 6 and 24 h. Co-IP was performed with an IgG antibody control and a FOXM1 antibody (αFOXM1); Inputs (1/10 of IP), and IP products with IgG and a FOXM1 antibody (αFOXM1) were resolved on western blot and probed for OTUB1. (**d**) Lysates prepared from epirubicin-treated MCF-7 and MCF-7Epi^R^ cells as in (**c**) were precipitated with an IgG antibody control, a FOXM1 (αFOXM1) and an OTUB1 (αOTUB1) antibody and probed for FOXM1 expression.

To confirm the mass spectrometry-based proteomic finding, we first examined whether endogenous OTUB1 binds to FOXM1 in the drug-sensitive MCF-7 and the drug-resistant MCF-7Epi^R^ cells by co-immunoprecipitation. In these cell extracts, we detected FOXM1 after immunoprecipitation with an OTUB1 antibody and vice versa, but not with the control IgG, indicating that OTUB1 interacts with FOXM1 either directly or as part of a larger complex. In addition, the co-immunoprecipitation studies also revealed that the FOXM1-OTUB1 complexes were more abundant in the MCF-7Epi^R^ compared with MCF-7 cells (Figures 1a and b and Supplementary Figure S1a). The FOXM1-OTUB1 interaction was further confirmed by the ability of FOXM1 to co-precipitate with a transiently transfected Flag-tagged OTUB1 (Supplementary Figure S1b). Epirubicin treatment caused a rapid decrease in the FOXM1-OTUB1 interaction in the sensitive MCF-7 cells but the levels of FOXM1-OTUB1 complexes were largely maintained or increased in the resistant MCF-7Epi^R^ cells (Figures 1c and d; also see Figure 3). Given that OTUB1 is a deubiquitinating enzyme that removes polyubiquitin chains and promotes stability of target proteins,^14, 15, 16^ these data suggest that OTUB1 binds to FOXM1 and modulates its ubiquitination and degradation in response to genotoxic treatment in breast cancer.

### OTUB1 regulates FOXM1 expression in response to epirubicin in breast cancer cells

**Figure 2:**
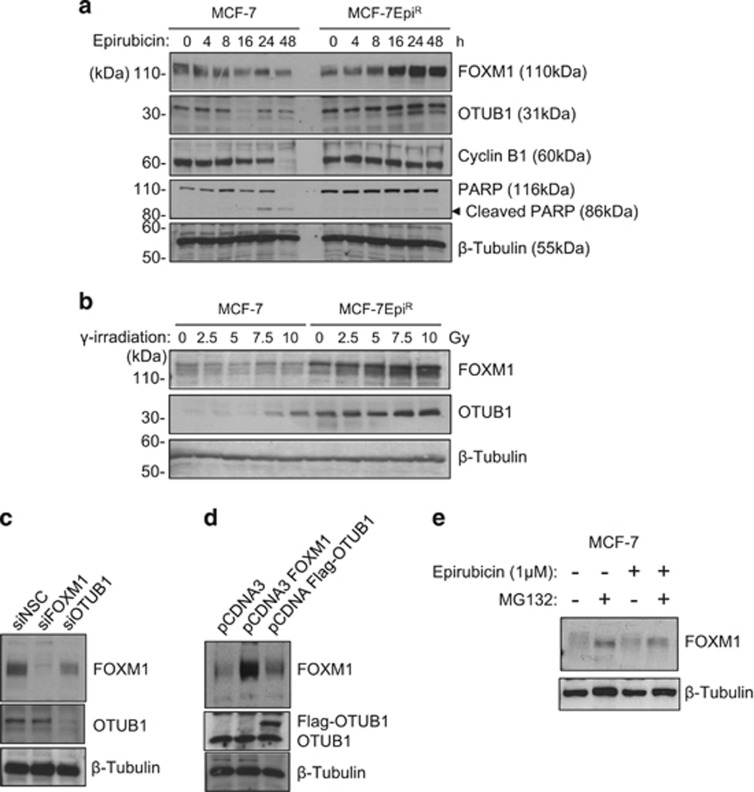
FOXM1 correlates with OTUB1 expression in MCF-7 and MCF-7Epi^R^ cells. (**a**) Protein expression levels of FOXM1, OTUB1, cyclin B1 and PARP in MCF-7 and MCF-7Epi^R^ cell lines were examined by western blotting after epiribicin treatment at different time points indicated. β-Tubulin was probed as a control for protein loading. Notably, there was cleavage of PARP in the sensitive and not resistant cells upon epirubicin treatment. (**b**) Protein expression levels of FOXM1 and OTUB1 in MCF-7 and MCF-7Epi^R^ cell lines were examined by western blotting with specific antibodies as indicated 24 h after treatment with different doses of γ-irradiation as indicated. (**c**) The expression levels of FOXM1, OTUB1 and β-tubulin were analysed by western blot analysis in MCF-7 cells transfected with the non-silencing control (NSC) siRNA, siRNA pool against FOXM1 or siRNA pool targeting OTUB1. (**d**) The expression levels of FOXM1, OTUB1 and β-tubulin were examined by western blot analysis in MCF-7 cells transfected with the empty expression vector pcDNA3, pcDNA3-FOXM1 or pcDNA3-Flag-tagged OTUB1. (**e**) The expression levels of FOXM1 and β-tubulin were examined by western blot analysis in MCF-7 cells treated with the proteasome inhibitor MG132 in the presence or the absence of epirubicin for 24h.

To explore the potential regulation of FOXM1 by OTUB1 in response to genotoxic agents, we investigated the expression of OTUB1 and FOXM1 in the epirubicin-sensitive MCF-7 and epirubicin-resistant MCF-7Epi^R^ breast carcinoma cell lines following epirubicin treatment. Western blot results showed that both OTUB1 and FOXM1 expressed at higher levels in the resistant MCF-7Epi^R^ cells compared with the parental MCF-7 cells. Moreover, both OTUB1 and FOXM1 decreased in expression levels in response to epirubicin in the sensitive cells, but were moderately induced in the resistant cells (Figure 2a). Consistently, the expression of the FOXM1 downstream target cyclin B1 also displayed a similar expression pattern as FOXM1 and OTUB1. Furthermore, epirubicin treatment also induced the accumulation of cleaved-PARP, indicative of apoptosis, in the sensitive MCF-7 but not in the resistant MCF-7Epi^R^ cells, confirming the differential drug sensitivity of these MCF-7 cells. Tubulin was also immunoblotted for as a control to ensure equal loading. Next, we studied the expression of OTUB1 and FOXM1 in response to another DNA-damaging agent, γ-irradiation. To this end, MCF-7 and MCF-7Epi^R^ cells were harvested for western blot analysis at 24 h after exposure to various doses of γ-irradiation (Figure 2b). The results again revealed that the expression levels of OTUB1 and FOXM1 were higher in the resistant compared with the sensitive MCF-7 cells. Although γ-irradiation caused an induction in OTUB1 and a corresponding increase in FOXM1 expression in a dose-dependent manner in the MCF-7Epi^R^ cells, the induction of OTUB1 by γ-irradiation were not matched by a similar induction of FOXM1 expression in the MCF-7 cells, which likely reflects the low baseline levels of OTUB1 in MCF-7 cells. Nevertheless, the correlations between the kinetics of OTUB1 and FOXM1 expression in the sensitive and resistant MCF-7 cells in response to DNA-damaging agents support the notion that OTUB1 restricts the downregulation of FOXM1 expression in response to DNA damage and genotoxic agents.

### FOXM1 expression is positively regulated by OTUB1 and negatively by the proteasome pathway

To investigate the idea that OTUB1 modulates FOXM1 expression further, we examined the effects of silencing OTUB1 and FOXM1 on the expression of endogenous OTUB1 and FOXM1 protein in MCF-7 cells. The results showed that in MCF-7 cells OTUB1 depletion culminated in the downregulation of FOXM1, but not vice versa (Figure 2c), suggesting that OTUB1 positively regulates FOXM1 expression. In agreement, OTUB1 overexpression also caused an increase in FOXM1 levels (Figure 2d). It is noteworthy that depletion of FOXM1 had little effect on OTUB1 mRNA levels (Supplementary Figure S2). Together these results suggest that OTUB1 regulates FOXM1 expression primarily at the translational or posttranslational levels (Figure 2d). The results also indicate that OTUB1 has a role in modulating the steady-state level of the FOXM1 protein.

Given that OTUB1 has been shown to restrict the ubiquitination and proteasome degradation of target proteins in response to DNA damage,^17^ we asked whether FOXM1 is also downregulated through protein degradation by the ubiquitin–proteasome pathway and tested the effect of the proteasome inhibitor MG132 treatment on the expression of endogenous FOXM1 in the absence or presence of epirubicin in MCF-7 cells (Figure 2e). Treatment with MG132 essentially prevented the downregulation in FOXM1 expression levels by epirubicin, indicating that the downregulation of FOXM1 expression in response to epirubicin is, at least partially, due to proteasomal degradation. It is also notable that MG132 enhanced the expression of FOXM1 in untreated cells, suggesting that FOXM1 is continuously downregulated by proteasomal degradation at the steady state level.

### OTUB1 binding promotes FOXM1 expression and attenuates Lys-48 linked ubiquitination in MCF-7 breast cancer cells

**Figure 3:**
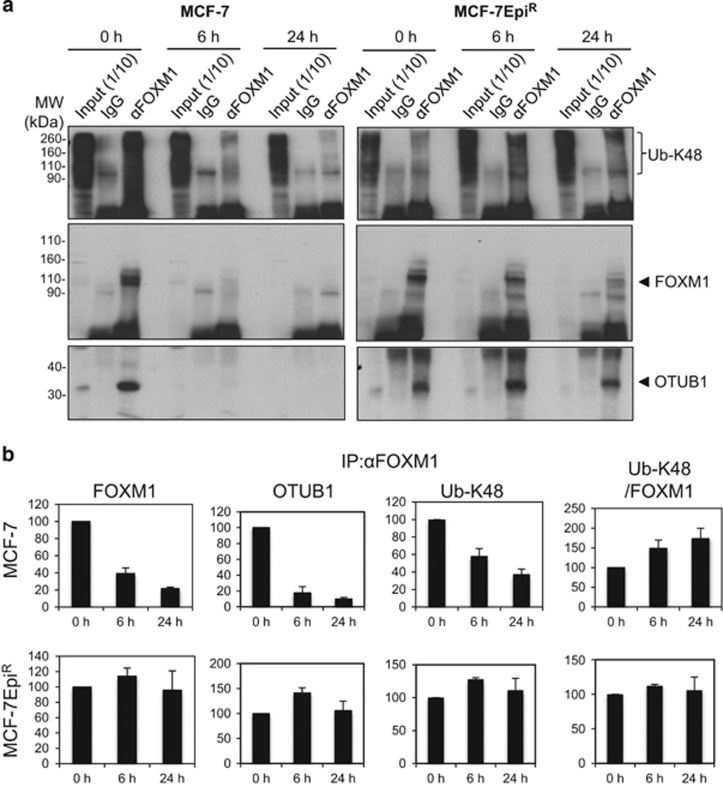
The downregulation of FOXM1 expression by epirubicin is associated with a decrease in OTUB1 binding and an increase in Lys48-linked polyubiquitin conjugates in MCF-7 cells. (**a**) Protein lysates prepared from MCF-7 and cells at 0, 6 and 24 h following treatment with 1 μM epirubicin were subjects to imnunoprecipitation with a FOXM1 antibody (αFOXM1). The Input and immunoprecipitates were then analysed by western blot analysis using antibodies against Lys48-linked ubiquitin, OTUB1 and FOXM1. Representative co-immunprecipitation results are shown. (**b**) The Lys48-linked polyubiquitin, OTUB1 and FOXM1 images were quantified using ImageJ analysis and plotted against signals at 0 h. The ratios of Lys48-polyubiquitin- conjugated FOXM1 relative to FOXM1 precipitates were also shown. Data shown represent the mean±s.d. from three independent experiments. (*t*-test: 6 h or 24 h versus 0 h epirubicin treatment; *significant *P*<0.05, **very significant *P*<0.01, and ns: not significant). ANOVA analysis was also performed on these data with *post hoc* test (Supplementary Figure S4).

We next examined whether OTUB1 binding is associated with the suppression of FOXM1 ubiquitination and downregulation in the epirubicin-sensitive and -resistant MCF-7 cells. To this end, we studied by co-immunoprecipitation the levels of OTUB1 and ubiquitination chains associated with FOXM1 in MCF-7 and MCF-7^R^ cells in response to epirubicin (Figure 3a and Supplementary Figure S3). The result showed that epirubicin treatment caused a prominent decrease in FOXM1 levels, which was associated with decreased interactions with OTUB1 in the sensitive MCF-7 cells. By contrast, FOXM1 protein levels and its association with OTUB1 remained relatively constant in the resistant cells upon epirubicin treatment. There was also an apparent loss of polyubiquitin chains in the FOXM1 complexes upon epirubicin treatment in the MCF-7 cells, but this is likely the result of a decline in endogenous FOXM1 levels upon epirubicin treatment. However, when we quantified the FOXM1 and Lys-48-linked polyubiquitin conjugates from the same immunoprecipitates, we found that the relative levels of Lys-48-linked polyubiquitin chains co-precipitated with FOXM1 actually increased with epirubicin treatment in MCF-7 cells (Figure 3b; Supplementary Figure S4). This induction in Lys48-linked polyubiquitination by epirubicin in MCF-7 cells was also associated with a decline in the FOXM1-OTUB1 interaction and FOXM1 expression in the MCF-7 cells. Quantification of the immunoblot signals indicated the kinetics for the disassociation of FOXM1-OTUB1 complexes occurred at a faster rate than the downregulation of FOXM1 expression, indicating that OTUB1 disassociation precedes FOXM1 ubiquitination and degradation in response to epirubicin in MCF-7 cells (Figure 3b). By comparison, the relative levels of total and Lys48-linked polyubiquitin chains, FOXM1 protein and FOXM1-OTUB1 complexes remained relatively constant in the resistant cells. It is noteworthy that the kinetics for Lys63-linked polyubiquitin downregulation was similar to that of FOXM1 expression, indicating that the Lys63-linked polyubiquitin chains are less likely to be involved in FOXM1 degradation in response to epirubicin (Supplementary Figure S5). These results are consistent with the notion that OTUB1 binds to FOXM1 and limits its ubiquitination and degradation in MCF-7 breast cancer cells.

### Polyubiquitin chains conjugated to FOXM1 are suppressed by OTUB1

**Figure 4:**
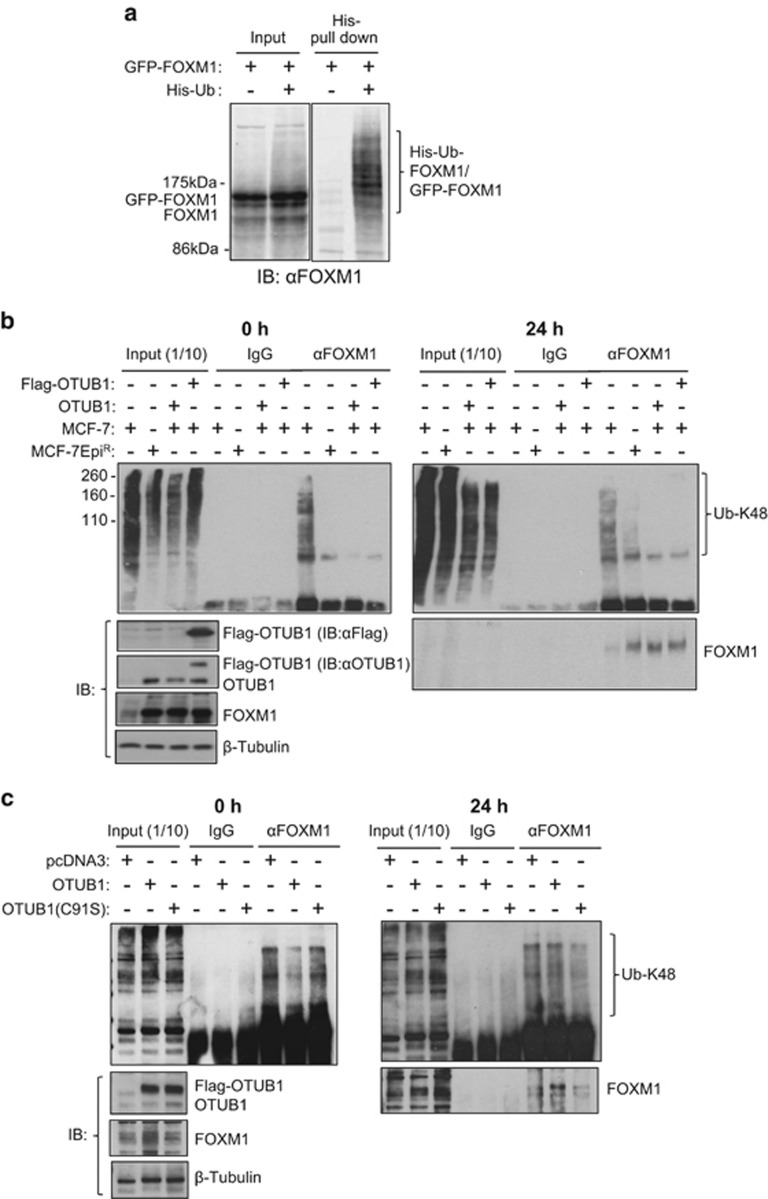
FOXM1 is modified by ubiquitination and its Lys48-linked polyubiquitin conjugates can be repressed by OTUB1. (**a**) MCF-7 cells were co-transfected with eGFP-FOXM1 and His-Ubiquitin. Ubiquitinated proteins were purified using Ni^2+^-column affinity pull-down under denaturing conditions (guanidinium chloride). Input and His-tagged Ubiquitin pulled-down proteins were probed for FOXM1 using an anti-FOXM1 antibody, and polyubiquitinated FOXM1 was detectable, demonstrating the covalent linkage of His-Ubiquitin to FOXM1. (**b**) MCF-7, MCF-7Epi^R^, and OTUB1 and Flag-OTUB1 stably expressing MCF-7 cells were treated with 1 μM epirubicin for 0 h or 24 h and 1 μM MG132 for 6 h and then subjected to immunoprecipitation with an FOXM1 antibody. Inputs (1/10 of IP) and IP products with control IgG and the FOXM1 antibody were resolved on western blot and probed for Lys48-linked polyubiquitin conjugates and FOXM1. The Inputs were also immunoblotted for FOXM1, OTUB1, β-tubulin and Flag-OTUB1 (anti-Fiag or αFlag) with the respective antibodies. (**c**) MCF-7 cells transfected with control pcDNA3, Flag-OTUB1(WT) or the Flag-tagged catalytically dead mutant OTUB1(C91S) were treated with 1 μM epirubicin for 0 h or 24 h, 10 μM MG132 for 6 h and then subjected to immunoprecipitation with an FOXM1 antibody and western blotted with specific antibodies as in (**c**).

We next investigated whether the polyubiquitin chains detected are directly conjugated to FOXM1. To confirm direct covalent linkage of Ubiquitin to FOXM1, we expressed a GFP-FOXM1 in the presence or absence of a His-tagged Ubiquitin, and pulled down the His-tagged ubiquitinated proteins under denaturing conditions after incubation with the proteasome inhibitor MG132 (Figure 4a). The purified His-tagged Ubiquitin-conjugated proteins were then immunoblotted with an anti-FOXM1 antibody, which detected the Ubiquitin-conjugated forms of FOXM1 as smears above the predicted GFP-FOXM1 molecular weight of 160kDa, indicative of polyubiquitin chains. We next studied by co-immunoprecipitation whether overexpression of OTUB1 can repress the conjugation of K48-linked polyubiquitin chains, which are known to promote target protein degradation, on FOXM1 in MCF-7 cells. The results showed that the FOXM1 immunoprecipitates contained higher levels of Lys48-linked polyubiquitin chains in both untreated and epirubicin-treated MCF-7 cells when compared with the epirubicin-resistant MCF-7Epi^R^ cells and two MCF-7 cell lines, one stably overexpressing OTUB1 and the other Flag-OTUB1 (Figure 4b and Supplementary Figure S6). The results also demonstrated that the levels of Lys48-linked polyubiquitin conjugates associated with FOXM1 increased upon epirubicin treatment. We next investigated whether the deubiquitinase activity of OTUB1 is required for FOXM1 stabilization and K48-polyubiquitin chain formation. To achieve this, we transfected MCF-7 with either the empty expression vector, wild-type (WT) OTUB1, or the OTUB1(C91S) mutant and studied the levels of K48-polyubiquitin chains associated with FOXM1 by immunoprecipitation (Figure4c). The FOXM1 co-immunoprecipitation analysis showed that overexpression of OTUB1, but not the OTUB1(C91S) mutant, substantially depleted the levels of K48-polyubiquitin chains and induced FOXM1 expression, particularly following epirubicin treatment (Figure 4c). These results suggest that the deubiquitinase activity of OTUB1 is required for the suppression of Lys48-linked FOXM1 polyubiquitination chains and for enhancing FOXM1 stability.

### OTUB1 enhances FOXM1 stability in response to epirubicin treatment

**Figure 5:**
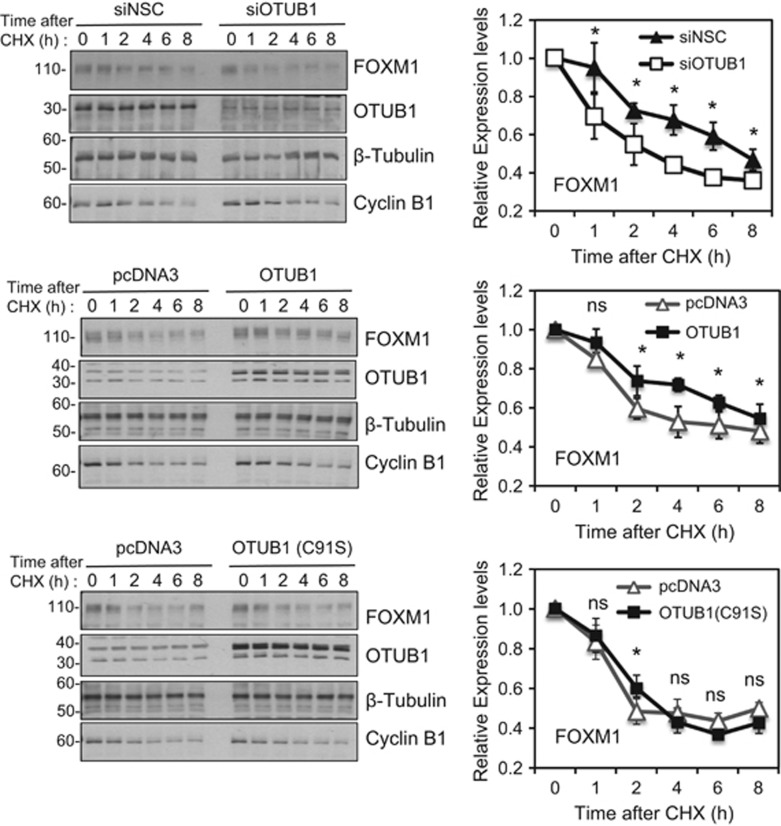
OTUB1 supresses the degradation of FOXM1 in epirubicin-treated MCF-7 cells. (**a**) MCF-7 cells transfected with control non-silencing siRNA or Smart Pool siRNA targeting OTUB1 were treated with 1 μM epirubicin for 16 h. These epirubicin-treated MCF-7 cells were then treated with cycloheximide, and protein lysates prepared from 0 to 8 h following cyclohexamide treatment. Protein expression levels of FOXM1, OTUB1, β-tubulin and cyclin B1 in these MCF-7 lysates were examined by western blotting. Densitometry was used to quantify the FOXM1 and β-tubulin levels from which independent background readings were subtracted. Western blots are representative of three independent experiments. The relative expression levels shown (right panels) are means±s.e.m. of the ratios of FOXM1 to β-tubulin levels relative to those at 0 h. Statistical significance was determined by Student's *t*-test (*P⩽0.05, significant; ns, non-significant). In (**b**) and (**c**), MCF-7 cells transfected with control pcDNA3 and (**b**) Flag-OTUB1(WT) or (**c**) the mutant Flag-OTUB1(C91S) were treated and analysed as in (**a**).

To examine the role of OTUB1 in regulating FOXM1 protein stability, MCF-7 cells were transfected with either control vector, OTUB1(WT) or OTUB1 (C91S, and subjected to treatment with epirubicin and then the translation inhibitor cycloheximide (Figure 5). Inhibition of *de novo* protein synthesis by cycloheximide caused a decline in FOXM1 protein levels in MCF-7 cells treated with epirubicin. Under these conditions, the rate of FOXM1 loss was reduced in MCF-7 cells transfected with OTUB1(WT) compared with cells transfected with the empty vector control. By contrast, the rates for the decline in FOXM1 levels were similar in MCF-7 cells transfected with the OTUB1(C91S) mutant and the empty vector. These findings indicate that the degradation of FOXM1 is impaired by overexpression of WT OTUB1 but not a mutant that lacks catalytic activity. Conversely, knockdown of OTUB1 by siRNA smart pool resulted in decreased half-life of FOXM1 in MCF-7 cells treated with epirubicin compared with cells transfected with non-silencing control siRNA. Together, these results suggest that the suppression of FOXM1 degradation by OTUB1 in response to epirubcin requires its deubiquitinating catalytic activity, further confirming that FOXM1 is a novel target of the deubiquitinase OTUB1. As an internal control, we measured the turnover of cyclin B1, a target of FOXM1 and not OTUB1, in response to epirubicin treatment. Taken together, the results provided strong evidence that OTUB1 suppresses FOXM1 ubiquitination and degraduation.

### OTUB1 promotes cell proliferation and epirubicin resistance through targeting FOXM1

**Figure 6:**
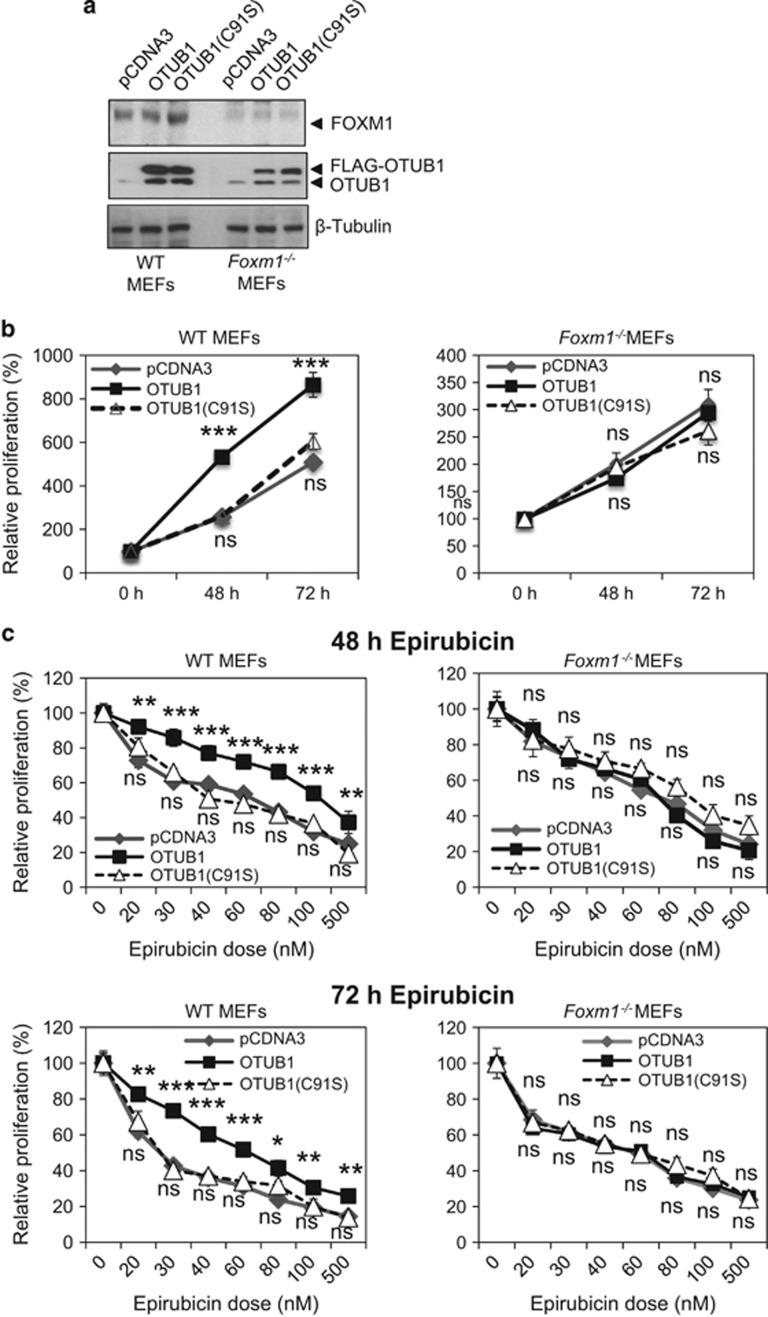
Overexpression of OTUB1 promotes cell proliferation, and epirubicin resistance in WT but not *Foxm1*-deficent MEFs. WT and *Foxm1*-deficent MEFs were transiently transfected with either the control pcDNA3 and Flag-OTUB1(WT) or the mutant Flag-OTUB1(C91S). (**a**) Twenty-four hours after transfection, protein lysates were prepared from these cells and then analysed for the expression of FOXM1, OTUB1 and β-Tubulin. (**b**) Twenty-four hours after transfection, aliquots of the transfected cells were split into 96 well plates and their proliferation analysed at the times indicated by SRB assays. Cell proliferation assays revealed that while untreated WT MEFs cells transiently transfected with Flag-OTUB1(WT) but not Flag-OTUB1(C91S), proliferated faster than the control pcDNA3-Flag cells, there was no difference in the *Foxm1*-deficent MEFs (c) The transfected cells were also treated with a range of doses of epirubicin (0-500 nM) and their proliferative rates assayed by SRB assay at 48 and 72 h after epirubicin treatment. Statistical significance was determined by Student's *t*-test (*P⩽0.05, **P⩽0.01, ***P⩽0.005; ns, non-significant) by comparing the proliferation rates of cells transfected with Flag-OTUB1(WT) or Flag-OTUB1(C91S) with the control pcDNA3-Flag transfected cells.

**Figure 7:**
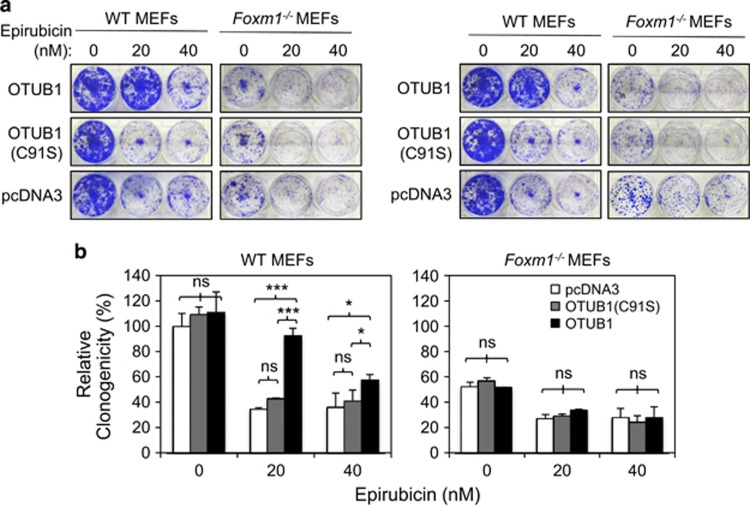
Overexpression of OTUB1 promotes clonogenicity in WT but not *Foxm1*-deficent MEFs. WT and *Foxm1*-deficent MEFs were transfected with either the control pcDNA3 and Flag-OTUB1(WT) or the mutant Flag-OTUB1(C91S). Twenty-four hours after transfection, the 2000 cells were seeded in six-well plates, treated with 0, 20 or 40 nM of epirubicn, grown for 15 days and then stained with crystal violet (top panel). The result (bottom panel) represents the average of three independent experiments±s.d. Statistical significance was determined by Student's *t*-test (*P⩽0.05, ***P⩽0.005; ns, non-significant).

Recent evidence demonstrates that FOXM1 can enhance cancer cell proliferation and protect cells from genotoxic agent-induced cell death by enhancing DNA damage repair.^5, 8, 12^ To test the possible function of OTUB1 in the regulation of breast cancer cell proliferation and DNA-damaging agent resistance, we transiently transfected MCF-7 cells with an OTUB1 expression plasmid and OTUB1 siRNA pool, and studied their effects on MCF-7 cell proliferation. The result showed that overexpression of OTUB1 significantly enhanced MCF-7 cell proliferation, whereas OTUB1 depletion decreased the rates of proliferation of MCF-7 cells (Supplementary Figure S7). Furthermore, we also found that OTUB1 overexpression enhanced the resistance of MCF-7 cells to epirubicin (Supplementary Figure S7). Conversely, OTUB1 silencing potentiated the anti-proliferative function of epirubicin in MCF-7 cells (Supplementary Figure S7). We next tested whether FOXM1 has a role in the cell-proliferative and epirubicin-resistant functions of OTUB1. To this end, we transfected WT and *Foxm1*^*−/−*^ mouse embryo fibroblasts (MEFs) with either the empty expression vector, OTUB1(WT), or the OTUB1(C91S) mutant and examined their effects on cell proliferation and epirubicin resistance by SRB assay. Overexpression of OTUB1, but not the OTUB1(C91S) mutant, significantly enhanced the cell proliferation as well as the viability of WT MEFs in response to epirubicin (Figure 6). By contrast, there were no significant differences in cell proliferation rates and epirubicin sensitivity in FOXM1-deficent MEFs transfected with vector control, OTUB1 and the OTUB1(C91S) mutant. In addition, clonogenic assays showed that at 0, 20 and 40 nM epirubicin, the colony formation capacity of WT MEFs transfected with WT OTUB1 was significantly enhanced when compared with MEFs transfected with empty expression vector or the OTUB1(C91S) mutant (Figure 7). Similar to the cell proliferation data, there were no appreciable differences in clonogenicity in *Foxm1*^*−/−*^ MEFs transfected with empty vector control, WT OTUB1 and the mutant OTUB1(C91S). These results indicate that FOXM1 is a key target of OTUB1 and that FOXM1 mediates the oncogenic and DNA damage resistant functions of OTUB1.

### Correlation between OTUB1 and FOXM1 expression in breast cancer patient samples

**Figure 8:**
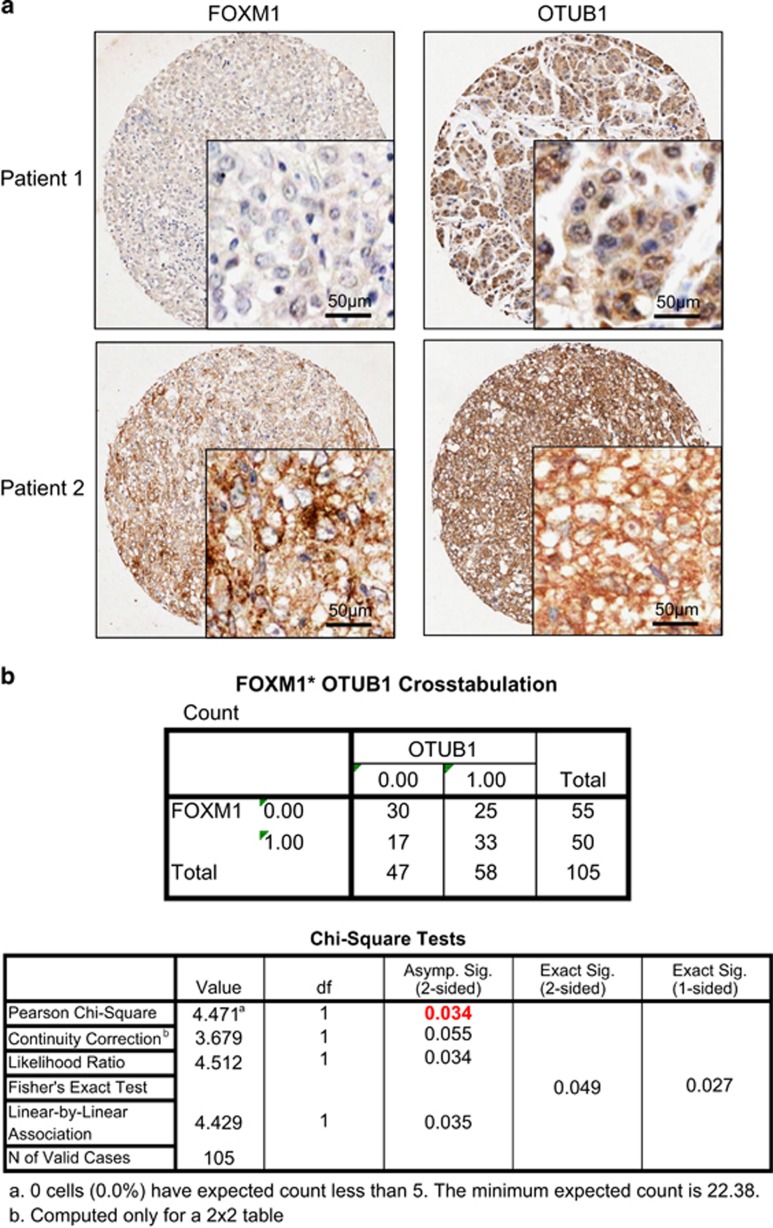
Positive correlation between FOXM1 and OTUB1 expression in breast cancer patients. (**a**) FOXM1 and OTUB1 expression was assessed by immunohistochemistry using tissue microarray constructed from 116 breast cancer patient samples. OTUB1 was expressed predominantly in the cytoplasm. Representative staining images of one patient with high FOXM1 and OTUb1 expression and one with low expression are shown. Images (magnification × 20; Insets (magnification × 100). Positive correlation between FOXM1 and OTUB1 expression was observed. (**b**) KIF20A staining were detected in both nuclear and cytoplasmic compartments and were correlated with FOXM1 staining. Statistical analysis revealed that OTUB1 were significantly correlated with FOXM1 expression (*P*= 0.034, Chi-Square test).

To establish further the physiological significance and clinical relevance of the regulation of FOXM1 by OTUB1 in breast cancer, FOXM1 and OTUB1 expression was assessed by immunohistochemistry in 116 breast cancer patient samples (Figure 8). OTUB1 was predominantly expressed in cytoplasm, consistent with its function as a deubiquitinating enzyme. Immunohistochemical analysis results showed that OTUB1 expression significantly correlated with FOXM1 expression (Chi-square test, *P*=0.034). There was also significant correlation between OTUB1 and oestrogen receptor (ER)α observed in our cohort (Supplementary Figure S8), consistent with the previous finding that OTUB1 could deubiquitinate and inhibit the degradation of ERα.^18^ However, there were no significant correlations between OTUB1 and other clinicopathological parameters including progesterone status, histological type, lymph node involvement and tumour stage (Supplementary Figure S8). Intriguingly, survival analysis showed no significant correlation between OTUB1 expression and patients' survival (Supplementary Figure S9). In agreement with our survival data, analysis of OTUB1 transcript expression in a previously published cohort (3455 breast cancer patients)^19^ also revealed that OTUB1 mRNA expression level was not significantly associated with poor survival (*P*=0.42 for overall survival, Kaplan–Meier analysis) (Supplementary Figure S10). However, in another published cohort of 1926 cases of lung cancer,^19^ OTUB1 mRNA expression level was a significant poor prognostic marker (*P*=0.0025 for overall survival, Kaplan–Meier analysis) (Supplementary Figure S11). The discrepancy between breast cancer and other cancer types is likely because OTUB1 substrates, in particular ERα, have a particularly essential role in breast cancer initiation, progression and treatment. Although ERα signalling promotes breast cancer proliferation, expression of ERα is also associated with good prognosis because these patients are likely to benefit from endocrine therapy. Moreover, in our tissue microarray cohort, tumour stage and lymph node involvement were significantly associated with poor survival (*P*=0.007 and *P*=0.017, respectively, for overall survival; *P*=0.001 and *P*=0.01, respectively, for disease-specific survival, refer to cox regression univariate analysis table) (Supplementary Figures S11 and S12). Therefore, to remove the interferences of the clinicopathological parameters on the survival analysis of OTUB1, multivariate analyses using Cox regression model were performed. In multivariate analysis, OTUB1 expression became significantly associated with poor survival after being adjusted for the clinicopathological parameters, including ER status, progesterone status, tumour stage, histological type and lymph-node involvement (*P*=0.033, relative risk=2.859 for overall survival and *P*=0.013, relative risk=4.048 for disease-specific survival) (Supplementary Figure S11). In this cohort, 60% of patients received chemotherapy. For this subgroup of patients, elevated OTUB1 was also significantly associated with poor survival after being adjusted for all the clinicopathological parameters (*P*=0.032, relative risk=3.822 for overall survival and *P*=0.032, relative risk=3.822 for disease-specific survival) (Supplementary Figure S12). As resistance to chemotherapy is known to be associated with poorer survival, the results from cox regression analysis supported a role of OTUB1 in chemotherapy resistance. This is consistent with our previous study showing that FOXM1 overexpression confers resistance to genotoxic chemotherapy.^9^ Collectively, these data suggest that OTUB1 overexpression is associated with chemotherapeutic drug resistance, and this is likely to be mediated by promoting FOXM1 protein expression through supressing its ubiquitination and degradation.

## Discussion

FOXM1 has an essential function in enhancing DNA damage repair and genotoxic agent resistance.^5, 8, 9, 10^ Previous research has also demonstrated that OTUB1 can modulate DNA damage response by promoting protein deubiquitination.^17^ We show, in here, that OTUB1 can promote deubiquitination and stabilization of FOXM1 in response to the genotoxic agent epirubicin in breast cancer.

Here, we also report that FOXM1 is conjugated with Lys48-linked polyubiquitin chains. FOXM1 expression is downregulated in response to epirubicin treatment in MCF-7 cells. This finding is consistent with the established concept that proteins with Lys48-linked polyubiquitin chains are generally targeted for proteasome-dependent degradation.^20, 21, 22, 23, 24, 25, 26, 27^ Different OTU family proteins have certain specificity towards distinct types of ubiquitin linkages.^28^ For example, OTUB1 preferentially targets polyubiquitin chains joined by Lys48 bonds, while OTUB2 has specificity towards chains associated through Lys63.^28^

In concordance, our data demonstrate that OTUB1 overexpression represses the conjugation of Lys48-linked polyubiquitin chains on FOXM1 and impairs its degradation, thus promoting resistance to genotoxic agents. Our previous study shows that SUMOylation inhibits FOXM1 activity, promotes its ubiquitination and enhances its cytoplasmic translocation as well as degradation.^10^ Thus, deubiquitination of FOXM1 may not only result in the stabilization of FOXM1 but may also promote its nuclear retention.^10^

In addition to its function as a deubiquitinase, previous studies have shown that OTUB1 can suppress ubiquitination by inhibiting the E2 ubiquitin-conjugating enzymes independently of its catalytic activity. For example, OTUB1 suppresses RNF168-dependent polyubiquitination independently of its catalytic activity. Accordingly, OTUB1 binds to and inhibits UBC13 (UBE2N), the cognate E2 enzyme for RNF168.^17^ OTUB1 also suppresses UBCH5 E2 enzyme and stabilizes the p53 protein.^14, 29^ However, we demonstrate here that OTUB1 suppresses the ubiquitination and degradation of FOXM1 through its deubiquitinase enzymatic activity. Our data show that WT OTUB1, but not the OTUB1(C91S) catalytic dead mutant, can enhance the stability of FOXM1 in response to epirubicin in MCF-7 cells, suggesting that the deubiquitinase activity is required for the degradation of FOXM1. The fact that OTUB1 interacts with FOXM1 also supports that FOXM1 deubiquitination occurs through a catalytic mechanism, that requires interactions with OTUB1.

The physiological relevance of the regulation of FOXM1 by OTUB1 is underscored by the strong and significant association between FOXM1 and OTUB1 in breast cancer patient samples. Despite not being an independent prognostic marker in Kaplan–Meier survival analysis, OTUB1 is significantly associated with poorer prognosis in multivariate regression analysis, particularly in breast cancer patients who have received chemotherapy. This argues that OTUB1 is likely to be linked to chemotherapy sensitivity in breast cancer. In agreement, the overexpression of OTUB1 has been shown to be associated with poorer outcome and cancer progression. For example, OTUB1 is associated with metastasis and is a predictive marker of poor prognosis in colorectal cancer.^30, 31^ In addition, OTUB1 promotes tumourigenesis and cell invasion in prostate cancer.^32^

Kaplan–Meier survival analysis shows that OTUB1 expression is not correlated with clinical outcome in breast cancer patients, even though OTUB1 is a predictor for prognosis in patients with other malignancies, such as colorectal cancer.^30, 31^ Similarly, despite OTUB1 mRNA level being a prognostic marker for lung cancer patients, its expression has no predictive value for patients diagnosed with breast cancer. This is likely to be due to the complex role played by OTUB1 in breast cancer, as it regulates the expression and activity of a variety of substrate proteins with diverse and sometimes opposing functions in breast cancer initiation, progression and treatment. OTUB1 may also affect the stability of proteins, which contribute towards breast cancer progression and treatment. For example, OTUB1 has been reported to be an ERα-interacting protein. OTUB1 can suppress ubiquitination and stabilize the expression of ERα in the chromatin and thereby, negatively regulate its transcription.^18^ In agreement, we also found a significant positive correlation between OTUB1 expression and ER status in our study (Supplementary Figure S8). While ERα signalling has a key role in stimulating initial breast cancer cell proliferation during tumourigenesis, the expression of ERα is also an indication of a good outcome in breast cancer patients as ERα-positive patients can often be treated with endocrine therapy.^33^ Another OTUB1 target in breast cancer is the tumour suppressor p53. In response to DNA-damaging agents, OTUB1 can stabilize and activate p53, leading to the inhibition of cell proliferation and apoptosis.^14, 29^ However, p53 expression and function is commonly lost during tumourigenesis and thus, the role of OTUB1 in the subsequent breast cancer progression and drug resistance will not be clear-cut.^34, 35^

The finding that overexpression of OTUB1 has a prominent role in promoting cell proliferation and drug resistance suggests that OTUB1 is a potent oncogene. This is further supported by the fact that overexpression of OTUB1 is associated with poorer prognosis in many malignancies.^30, 31, 32^ However, except for ERα, few oncogenes have been identified to be substrates of OTUB1.^18^ In here, we not only show that the potent oncogene FOXM1 is a target of OTUB1 but also present evidence to demonstrate that the oncogenic and genotoxic functions of OTUB1 depend on the expression of a functional FOXM1, suggesting that OTUB1 promotes cell proliferation and epirubicin resistance predominantly through targeting FOXM1.

Collectively, these results provide strong evidence to suggest that not only OTUB1 deubiquitinase activity prevents the proteasomal degradation of FOXM1 upon epirubicin treatment, OTUB1 also regulates steady-state expression of FOXM1. Consistent with the emerging oncogenic role of OTUB1 in cancer, the proliferative and clonogenic assays also show that OTUB1 can enhance the proliferative rate and genotoxic agent resistance of breast cancer cells and that FOXM1 is a crucial substrate of OTUB1 in cancer cell proliferation and genotoxic agent resistance.

## Materials and methods

### Cell culture, plasmids and transfection reagents

The MCF-7 cell line used originated from the American Type Culture Collection and was acquired through CRUK cell bank (London, UK). MCF-7Epi^R^ cells and MEFs have previously been described.^8, 9^ Cells were cultured in DMEM (Sigma-Aldrich, Poole, UK) supplemented with 10% (v/v) feotal calf serum and 2 mM glutamine at 37 °C. Epirubicin Hydrochloride (2 mg/ml (3.4 mM) in 0.9% sodium chloride, Medac, Germany) was obtained from Imperial College Healthcare (London, UK). The pcDNA3-Flag-OTUB1 and -OTUB1(C91S) plasmids used in this study have previously been described.^17^ The eGFP-FOXM1 and pcDNA3-FOXM1 expression plasmids have also been described.^10^ For ubiquitination studies, cells were treated with 10 μM MG132 (M7449; Sigma-Aldrich) for 6 h before collection for analysis. Cells were transfected using FuGENE 6 transfection reagent (Promega, Southampton, UK) and XtremeGENE HP reagent (Roche Diagnostics, Welwyn Garden City, UK) as recommended by the manufacturers.

### Ni-NTA pull-down assays

His-tagged proteins were purified by nickel magnetic agarose beads (Qiagen, Manchester, UK) under denaturing conditions as described.^36^ For details, see also Supplementary Materials and Methods.

### Quantitative real-time PCR (qRT–PCR)

Total RNA was extracted with the RNeasy Mini Kit (Qiagen). Complementary DNA generated by Superscript III reverse transcriptase and oligo-dT primers (Invitrogen, Paisley, UK) was analysed by qRT–PCR as described.^8, 12^ See also Supplementary Materials and Methods.

### Gene silencing with siRNAs

For gene silencing, cells were transiently transfected with siRNA SMARTpool reagents purchased from Thermo Scientific Dharmacon (Lafayette, CO, USA) using Oligofectamine (Invitrogen, Life Technologies Ltd, Paisley, UK) according to the manufacturer's instructions. siRNAs On Target Smart Pool used were: siRNA FOXM1 (L-009762-00), siRNA OTUB1 (L-021061-00) and the non-silencing control siRNA (D-001810-10-05).

### Measure of FOXM1 protein turnover

The turnover rate of endogenous FOXM1 in MCF-7 cells was determined using cycloheximide (01810; Sigma-Aldrich) inhibition of protein synthesis. For details, see Supplementary Materials and Methods.

### Immunoprecipitation and western blotting

Western blotting was performed on whole-cell extracts by lysing cells in buffer or precipitates in the presence of N-ethyl-amide (10 mM) (Sigma UK, Poole, UK) as previously described.^9, 37^ See Supplementary Materials and Methods for antibodies used.

### Clonogenic assay

Clonogenic assay has been described.^9^ Also, see Supplementary Methods and Materials.

### Tissue microarray, immunohistochemistry and staining scoring

These reagents and analyses have been described.^9^ For details, see Supplementary Methods and Materials.

### Statistical analysis

All statistics were determined using SPSS 16.0 and Windows XP, Excel (Imperial College London, Software Shop, UK). Also, see Supplementary Methods and Materials.

## Supplementary Material

Supplementary Informations
