# Supplementary material for: OTUB1 inhibits the ubiquitination and degradation of FOXM1 in breast cancer and epirubicin resistance
Source: Oncogene. 2015 Jul 6;35(11):1433–44. doi: 10.1038/onc.2015.208 (PMC4606987; doi:10.1038/onc.2015.208)
Supplement: Supplementary Informations [file onc2015208x1.doc]

**Supplementary Materials and Methods**

***Western blotting and antibodies***

The antibodies against FOXM1 (C-20), β-tubulin (H-235), and Cyclin B1, were purchased from Santa Cruz Biotechnology (Santa Cruz, CA, USA). The K63- and K48-linkage specific polyubiquitin antibodies (D7A11 and 4289) were purchased from Cell Signaling Technology (New England Biolabs Ltd. Hitchin, UK). Antibodies against Ubiquitin (clone Ubi-1) and OTUB1 (O9889) were from Millipore (U.K.) Limited, (Watford, UK), and Sigma-Aldrich, respectively. Primary antibodies were detected using horseradish peroxidase-linked anti-mouse or anti-rabbit conjugates as appropriate (Dako, Glostrup, Denmark) and visualized using the ECL detection system (Amersham Biosciences, Pollards Wood, UK).

For co-immunoprecipitation experiments, cell lysates were prepared in IP buffer (1% Nonidet P-40, 150 mM NaCl, 50 mM Tris-HCl [pH 7.4], 10 mM NaF, 1 mM sodium orthovanadate, 10 mM N-ethyl-amide (NEM) and protease inhibitors [Complete protease inhibitor cocktail; Roche, Lewes, UK]) and pre-cleared lysate was immunoprecipitated with the indicated antibodies and protein A/G-sepharose for 2 h. Sepharose beads were then washed in lysis buffer and boiled for 5 min prior to being centrifuged (3500x *g*; 3 min). For cell fractionation MCF-7 cells were lysed in buffer A (10 mM HEPES pH 7.4, 10 mM KCl, 0.1 mM EDTA, 0.1 mM EGTA, 2mM DTT) and protease and phosphatase inhibitors, and incubated for 20 min on ice. NP-40 was added (final concentration 1%v/v) and centrifuged. The supernatant containing the cytoplasmic fraction was then frozen at -70^o^C. The pellet was washed in buffer A and resuspended in buffer B (10 mM HEPES, 10 mM KCl, 0.1mM EDTA, 0.1 mM EGTA, 2 mM DTT, 400 mM NaCl, 1% NP-40), and rotated at 4^o^C for 15 min. Samples were centrifuged at 4^o^C and the supernatent containing the nuclear extract collected and frozen at -70^o^C. Both buffer A and buffer B contained protease and phosphatase inhibitors. Proteins were separated by SDS–PAGE gel electrophoresis, transferred to Hybond-C membranes and immunoblotted with the indicated antibodies.

***Ni-NTA pull-down assays***

Ni-NTA pull down assays were performed by incubating Ni-NTA magnetic agarose beads (Qiagen, Manchester, UK) with appropriate HeLa cell lysates overnight in buffer A containing 10 mM imidazole, 6 M guanidinium chloride, 100 mM NaH_2_PO4 (pH. 8), 0.05% Tween20. The Ni-NTA beads were washed five times with buffer A and then 3 times with buffer B consisting of 8 M urea, 100 mM NaH_2_PO4 (pH 5.9), 0.05% Tween20 containing 10 mM imidazole, 20 mM Tris (pH 7.5), 150 mM NaCl, 2 mM EDTA and 0.05% (v/v) Triton X-100. Magnetic agarose-bound proteins were separated by SDS-PAGE and Western blots were visualized by ECL.

**Quantitative real-time PCR (qRT–PCR)**

Total RNA was extracted with the RNeasy Mini Kit (Qiagen). Complementary DNA generated by Superscript III reverse transcriptase and oligo-dT primers (Invitrogen, Paisley, UK) was analysed by qRT–PCR as described . See also Supplementary Materials and Methods. Transcript levels were quantified using the standard curve method. The following gene-specific primers were used: L19-sense: 5′-GCGGAAGGGTACAGCCAAT-3′ and L19-antisense: 5′-GCAGCCGGCGCAAA-3′; FOXM1-sense: 5′-TCCTCCACCCCGAGCAA-3′ and FOXM1-antisense: 5′-CGTGAGCCTCCAGGATTCAG-3′; OTUB1-sense: 5′- CAGGCCTGACGGCAACTG-3′ and OTUB1-antisense: 5′- AGTGCCTCCAAGTGGGAGAA -3′.

***Measure of FOXM1 protein turnover***

The turnover rate of endogenous FOXM1 in MCF-7 cells was determined using cycloheximide (CHX) (01810; Sigma-Aldrich) inhibition of protein synthesis. MCF-7 were transiently transfected with either pcDNA empty expression vector, or wild-type (WT) or C91S mutant OTUB1. Twenty-four hours after transfection CHX was added to the culture media to a final concentration of 80 μg/ml. Cells were harvested at indicated time points, and equal amounts of cell lysates were subjected to SDS-PAGE and analysed by immunoblotting (IB).

***Clonogenic Assay***

A total 2,000 cells were seeded into six-well plates and incubated overnight. The cells were then treated for 48 h with varying concentrations of paclitaxel (Teva UK Limited, East Sussex, UK). The drug was removed and surviving cells were left to form colonies. After 14 days of incubation, colonies were fixed with 4% Paraformaldehyde for fifteen minutes at room temperature and then washed with phosphate buffered saline. 0.5% crystal violet was used to stain the fixed cells for thirty minutes, following which the plates were washed with tap water. Plates were then left to dry overnight. Quantification was achieved by solubilising dye with 33% acetic acid and the absorbance was measured at 492nm using a microplate reader (Sunrise, Tecan, CA, USA).

***Tissue Microarray***

One hundred and thirty-three cases of breast cancer diagnosed between the years 1992 to 2001 with clinical follow up data were retrieved from the records of the Department of Pathology, Queen Mary Hospital of Hong Kong, with approval by the Institutional Review Board of The University of Hong Kong. Histological sections of all cases were reviewed by the pathologist, the representative paraffin tumour blocks chosen as donor block for each case and the selected areas marked for construction of tissue microarray (TMA) blocks. A total of 116 could be assessed and scored for FOXM1 and OTUB1 staining. The expression pattern and subcellular localization were correlated with various clinicopathological data including ER, PR status, age, histological grade, histological type, clinical stage, lymph node metastasis as well as survival time.

***Immunohistochemistry***

The TMA sections were deparaffinized and rehydrated by incubation with xylene and decreasing concentrations of ethanol. Citrate buffer (0.01M, pH 6.0) was used for antigen retrieval. The slides were immersed into 3% H_2_O_2_/methanol for 10 min at room temperature to quench endogenous peroxidase. After rinsing in 0.05% Tween in PBS (PBST) twice, FOXM1 (c-20; Santa Cruz, USA) and OTUB1 (O9889; Millipore UK) specific antibodies diluted at 1:1100 and 1:50 respectively, were added to each section and incubated at 4°C overnight. The slides were then washed in PBST and incubated with DAKO EnVision+System-HRP-labelled Polymer Anti-Rabbit at room temperature in dark for 30 min. After washing, Chromogen DAB/substrate reagent was added onto the slides and the slides incubated for a further 6 minutes. Finally, the slides were dehydrated and mounted. Aperio ScanScope ® system (Aperio technology, USA) was used to visualize and assess for protein expression.

***Staining scoring***

The stained TMA slides were scanned by ScanScope scanners and individual stained TMA spots were assessed in computer screen with the use of Aperio’s image viewer, ImageScope. To avoid subjectivity in evaluation, the intensities and percentages of the staining were scored by two independent individuals in a semi-quantitative way as previously described and average was taken {Chen, 2010 #407}. For each case, a final score was obtained by multiplying the score of intensity with the score of percentage, 12 being the maximum final score.

***Statistical analysis***

The correlation between FOXM1 and OTUB1 expression in TMA was assessed by bivariate Pearson Correlation analysis. The correlation between OTUB1 expression and patients’ survival was estimated by Kaplan-Meier estimation and compared by Log-rank test. Multivariate analysis was done by Cox-regression model. Where appropriate a two-tailed independent sample t-test was performed to analyse significance. *P* value less than 0.05 was considered to be statistically significant. For statistical analysis of three or more groups, a one way ANOVA with post-hoc test would be performed
