# Supplementary material for: OTUB1 inhibits the ubiquitination and degradation of FOXM1 in breast cancer and epirubicin resistance
Source: Oncogene. 2015 Jul 6;35(11):1433–44. doi: 10.1038/onc.2015.208 (PMC4606987; doi:10.1038/onc.2015.208)
Supplement: Supplementary Figure Legends [file onc2015208x2.doc]

**Supplementary Figure Legends**

**Supplementary Figure S1. FOXM1 complexes with OTUB1 in MCF-7 and MCF-7Epi^R^ cells. a)** Co-immunoprecipitation (co-IP) was performed with an IgG antibody control, a FOXM1 or an OTUB1 antibody on lysates from MCF-7 and MCF-7EpiR cells; Inputs (1/20 of IP), and IP products with IgG and specific antibodies were resolved on western blot and probed for FOXM1. FOXM1* represents a FOXM1 species associated with its SUMOylation (Myatt *et al*, 2014 Oncogene 33:4316-29. **b)** MCF-7 cells were transfected with the control pcDNA3-Flag vector and pcDNA3-Flag-OTUB1. Twenty-four hours later, the transfected cell lysates were immunoprecipitated with a FOXM1 antibody and immunoblotted with a Flag antibody (M2; Sigma Aldrich UK). The result shows OTUB1 complexes with FOXM1.

**Supplementary Figure S2. Quantitative RT-PCR analysis determining the relative mRNA expression levels of FOXM1 and OTUB1 in MCF-7 cells after FOXM1 and OTUB1 silencing**. The expression levels of FOXM1, OTUB1 and β-tubulin were analysed by qRT-PCR analysis in MCF-7 after transfection with the non-silencing control (NSC) siRNA, siRNA pool against FOXM1, or siRNA pool targeting OTUB1. Mean values and the ±S.D. of three experiments are shown (right panel). Student’s t-test was applied to determine statistical significance, **P ≤ 0.01; ***P ≤ 0.005.

**Supplementary Figure S3. OTUB1 complexes with FOXM1 in MCF-7 and MCF-7Epi^R^ cells.** MCF-7 and MCF-7Epi^R^ cells were treated with epirubicin (1μM) for 0, 6 and 24 h. Co-IP was performed with an IgG antibody control and a FOXM1 antibody; Inputs (1/10 of IP), and IP products with IgG and a FOXM1 antibody were resolved on western blot and probed for OTUB1 using a mouse and a rabbit OTUB1 antibody.

Asterisks show the positions of OTUB1.

**Supplementary Figure S4**. **Anova analysis of co-immunoprecipitation results**. For MCF-7 cells, there is significant difference for FOXM1, K48 and K48/FOXM1 tested by Anova. For MCF-7Epi^R^ cells, there is no significant difference for K48 and K48/FOXM1. However, for OTUB1 in MCF-7 and MCF-7Epi^R^ cells and FOXM1 in MCF-7Epi^R^ cells, the assumption of homogeneity of variance has been violated ; therefore, Anova test is no longer an accurate measurement. The alternative Welch test cannot be performed because the 0 h data has variance that equals to 0.

For these three sets of data, another post-hoc test (Games-Howell) which does not assume the equal variance, was used to compare the difference between groups. There was still significant differences between groups for OUTB1 in MCF7 and MCF-7Epi^R^ but no difference for FOXM1 in MCF-7Epi^R^.

**Supplementary Figure S5. The downregulation of FOXM1 expression by epirubicin is associated with a decrease in total and Lys63-linked polyubiquitin conjugates in MCF-7 cells. (a)** Protein lysates prepared from MCF-7 and MCF-7Epi^R^ cells at 0, 6 and 24 h following treatment with 1 μM epirubicin were subjects to imnunoprecipitation with a FOXM1 antibody. The Input and immunoprecipitates were then analysed by western blot analysis using antibodies against total Ubiquitin and Lys63-linked Ubiquitin. Representative co-immunprecipitation results are shown. **(b)** The images of the total polyubiquitin and Lys63-linked polyubiquitin conjugates were quantified using ImageJ® analysis and plotted against signals at 0 h. Data shown represent the mean ± SD from 3 independent experiments. (*t*-test: 6 h or 24 h verses 0 h epirubicin treatment; *significant *p*<0.05, ** very significant *p*<0.01, and ns: not significant). Notably, the total polyubiquitin conjugates decreased at a similar rate as the Lys48-linked polyubiquitin conjugates, while the Lys63-linked polyubiquitin conjugates were down-regulated at a faster kinetics.

**Supplementary Figure S6. Epirubicin induces and OTUB1 suppresses the formation of polyubiquitin conjugates associated with FOXM1 in MCF-7 cells.** MCF-7 cells were treated with epirubicin (1μM) for 0, 6 and 24 h with and without OTUB1 transfection. Co-IP was performed with an IgG antibody control and a FOXM1 antibody; Inputs (1/10 of IP), and IP products with IgG and a FOXM1 antibody were resolved on western blot and probed analysed using an antibody against Lys63-linked Ubiquitin.

**Supplementary Figure S7. Overexpression of OTUB1 promotes cell proliferation, and epirubicin resistance `in MCF-7 cells.**

MCF-7 breast cancer cells were transiently transfected with either the control pcDNA3 and Flag-OTUB1(WT) or the mutant Flag-OTUB1(C91S). (a) Twenty-four hours after transfection aliquots of the transfected cells were split into 96 well plates and their proliferation analysed at the times indicated by SRB assays. Cell proliferation assays revealed that while untreated MCF-7 cells transiently transfected with Flag-OTUB1(WT) but not Flag-OTUB1(C91S), grew faster than the control pcDNA-Flag cells. (c) The transfected cells were also treated with a range of doses of epirubicin (0-20 μM nM) and their proliferative rates assayed by SRB assay at 48 and 72 h after treatement. The result represents average of >3 independent experiments ± SD. Statistical significance was determined by Student’s t-test (*P≤0.05, **P≤0.01, ***P≤0.005; n.s., non-significant).

**Supplementary Figure S8. Correlation between OTUB1 and various clinicopathological parameters. a)** Representative negative staining control image of one patient. **b)** High OTUB1 expression significantly correlates with ER positivity (P= 0.035, Chi-Square test). OTUB1 expression is not associated with other clinicopathologcial parameters.

**Supplementary Figure S9. Kaplan-Meier analysis showing OTUB1 expression alone was not significantly correlated with patients’ survival.**

**Supplementary Figure S10** (a) OTUB1 transcript expression was not significantly associated with poor survival in a previously published cohort (3455 breast cancer patients) (Log-rank test, P=0.42 for overall survival). (b) OTUB1 mRNA expression was a significant poor prognostic marker in another published cohort of 1926 cases of lung cancer (Log-rank test, P=0.0025 for overall survival).

**Supplementary Figure S11.** **Cox regression analysis of all patients.** Multivariate analyses using Cox regression model showed that, after being adjusted for the clinicopathological parameters including ER status, PR status, tumour stage, histological type and lymph-node involvement, OTUB1 expression was significantly associated with poor survival (P = 0.033, RR=2.859 for overall survival and P=0.013, RR=4.048 for disease-specific survival, respectively).

**Supplementary Figure S12**. **Cox regression analysis of patients who received chemotherapy**. For the patients who received chemotherapy (n=60), elevated OTUB1 was significantly associated with poor survival after being adjusted for all the clinicopathological parameters by cox regression analysis (P=0.032, RR=3.822 for overall survival and P=0.032, RR=3.822 for disease-specific survival, respectively).
