# Supplementary material for: OTUB1 inhibits the ubiquitination and degradation of FOXM1 in breast cancer and epirubicin resistance
Source: Oncogene. 2015 Jul 6;35(11):1433–44. doi: 10.1038/onc.2015.208 (PMC4606987; doi:10.1038/onc.2015.208)
Supplement: Supplementary Figure S1 [file onc2015208x3.ppt]

## Slide 1
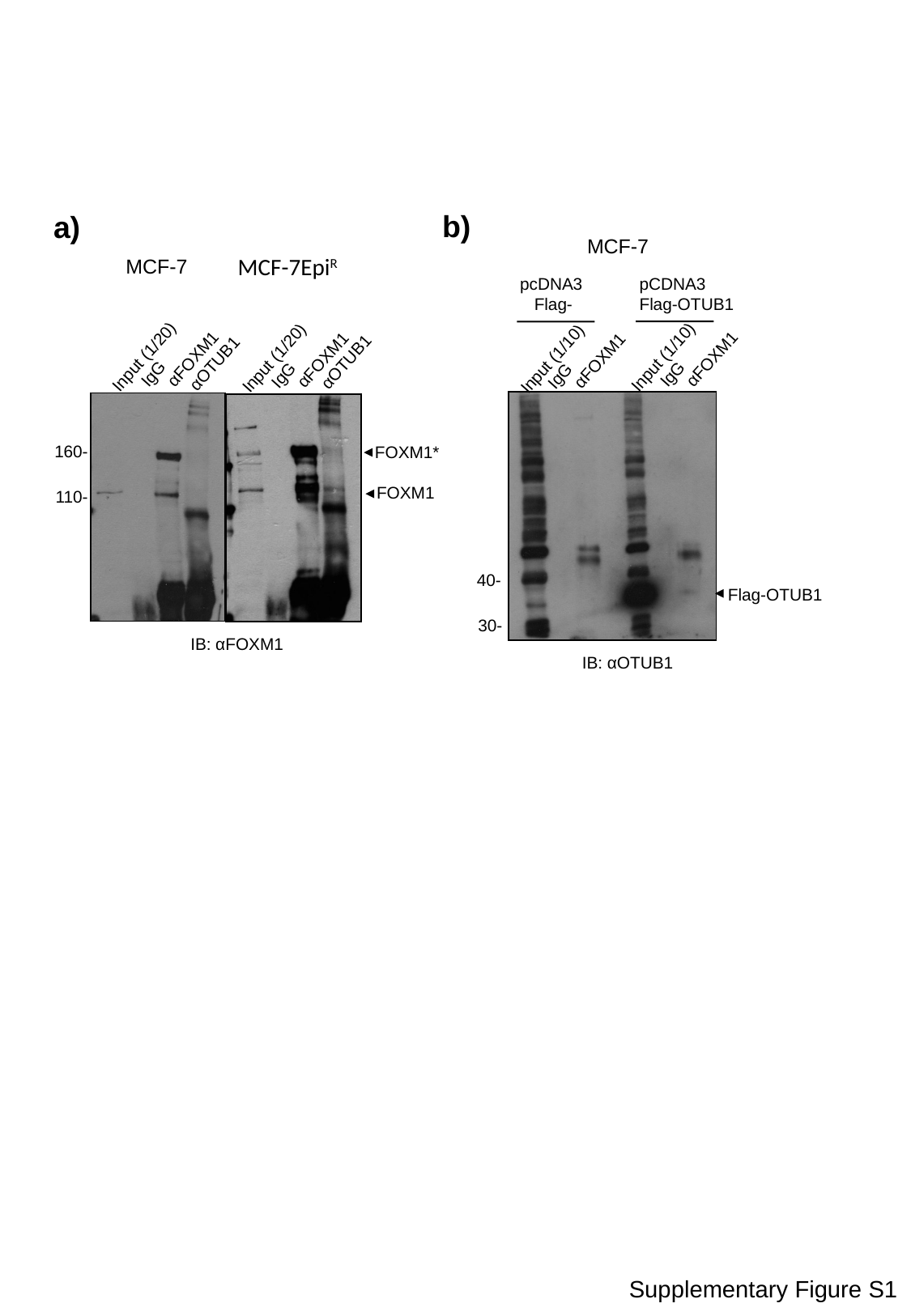

b)
a)
MCF-7
MCF-7EpiR
MCF-7
pcDNA3
Flag-
pCDNA3
Flag-OTUB1
Input (1/20)
Input (1/10)
Input (1/20)
αFOXM1
Input (1/10)
αFOXM1
αFOXM1
αFOXM1
αOTUB1
αOTUB1
IgG
IgG
IgG
IgG
160-
FOXM1*
FOXM1
110-
40-
Flag-OTUB1
30-
IB: αFOXM1
IB: αOTUB1
Supplementary Figure S1
