# Supplementary material for: OTUB1 inhibits the ubiquitination and degradation of FOXM1 in breast cancer and epirubicin resistance
Source: Oncogene. 2015 Jul 6;35(11):1433–44. doi: 10.1038/onc.2015.208 (PMC4606987; doi:10.1038/onc.2015.208)
Supplement: Supplementary Figure S2 [file onc2015208x4.ppt]

## Slide 1
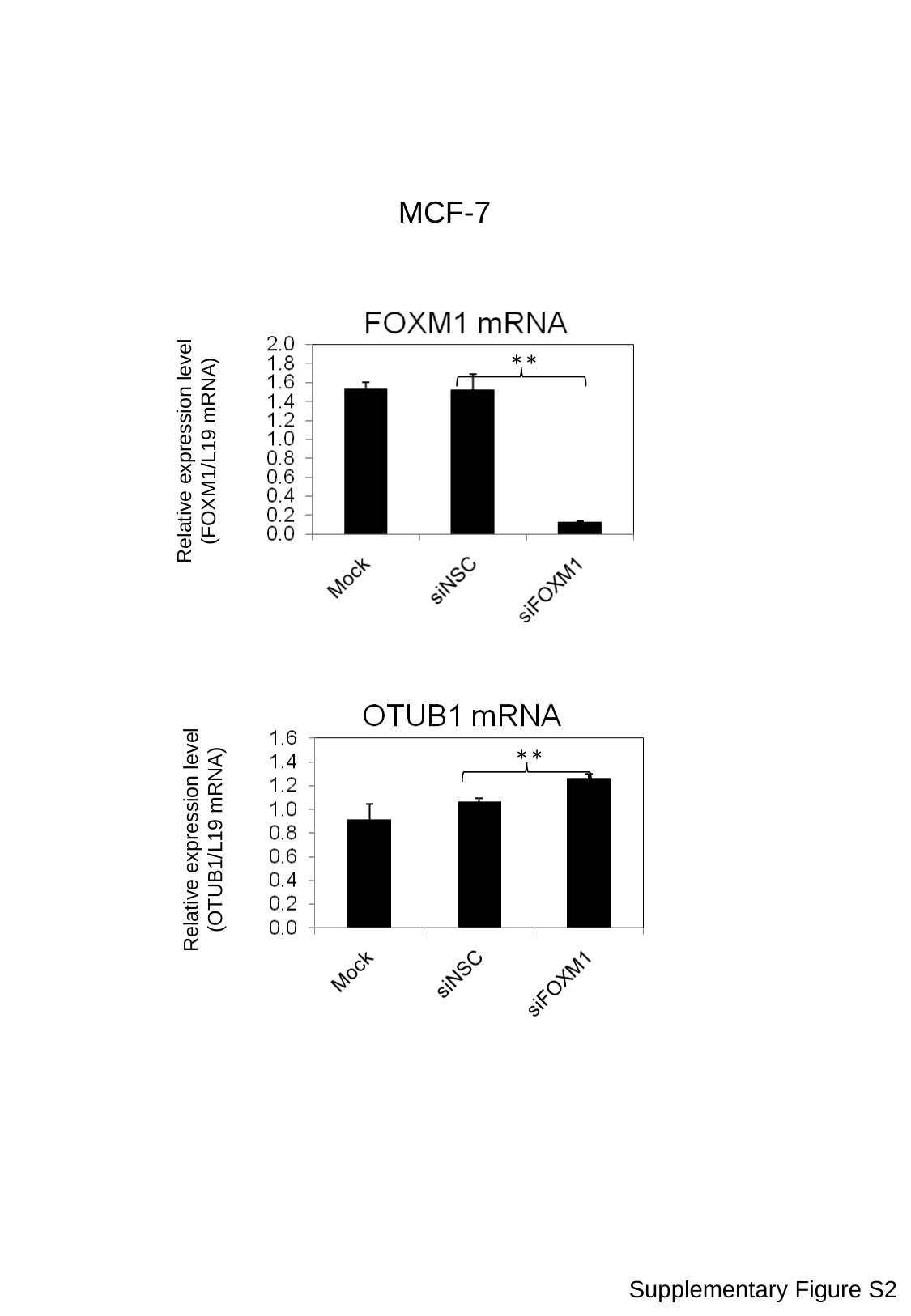

MCF-7
**
Relative expression level
(FOXM1/L19 mRNA)
**
Relative expression level
(OTUB1/L19 mRNA)
Supplementary Figure S2
