# Supplementary material for: OTUB1 inhibits the ubiquitination and degradation of FOXM1 in breast cancer and epirubicin resistance
Source: Oncogene. 2015 Jul 6;35(11):1433–44. doi: 10.1038/onc.2015.208 (PMC4606987; doi:10.1038/onc.2015.208)
Supplement: Supplementary Figure S3 [file onc2015208x5.ppt]

## Slide 1
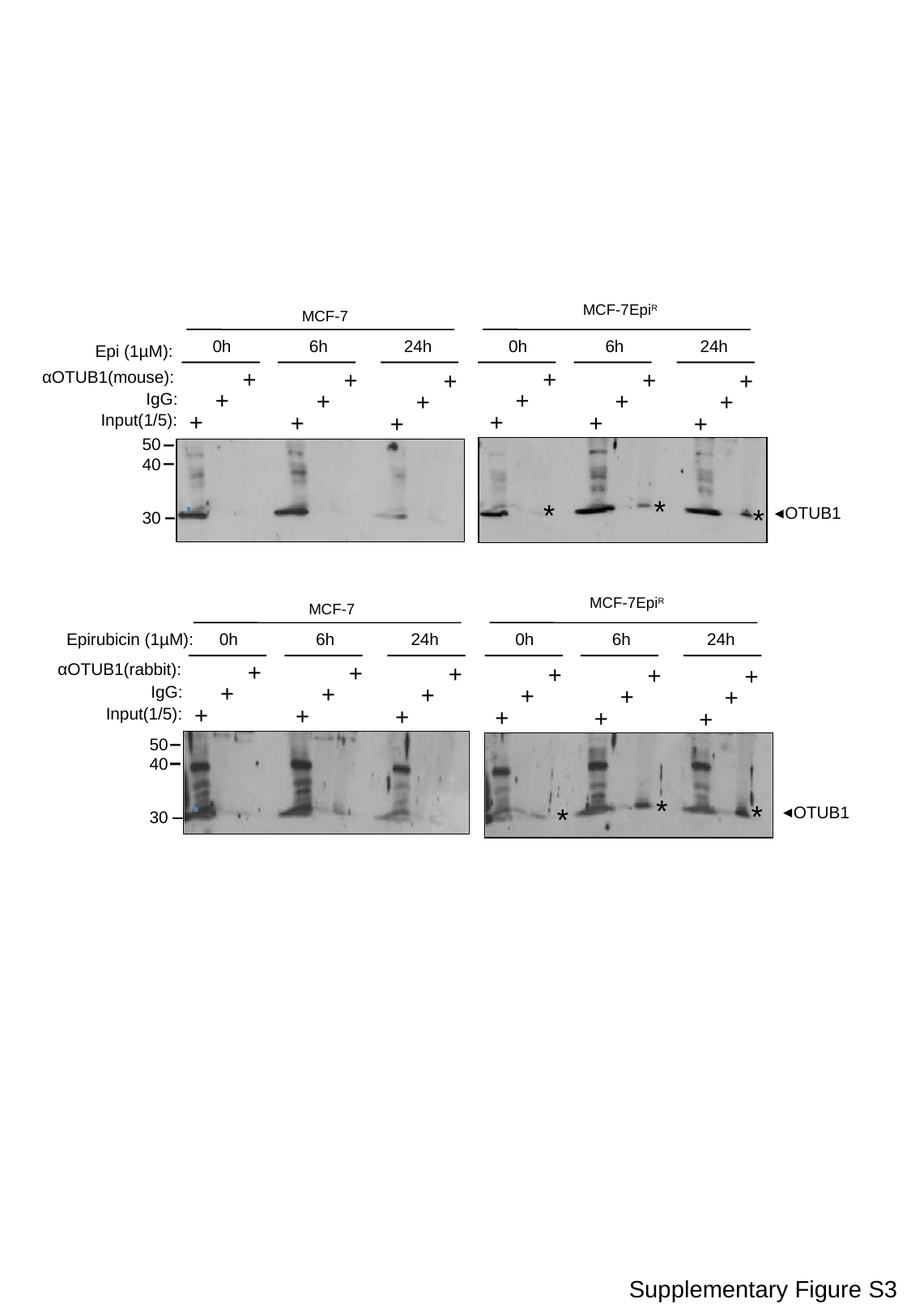

MCF-7EpiR
MCF-7
0h
6h
24h
0h
6h
24h
Epi (1µM):
+
+
+
+
+
+
αOTUB1(mouse):
+
+
+
+
+
+
IgG:
+
+
+
+
+
+
Input(1/5):
50
40
*
*
MCF-7EpiR
MCF-7
0h
6h
24h
0h
6h
24h
Epirubicin (1µM):
+
+
αOTUB1(rabbit):
+
+
+
+
+
+
+
IgG:
+
+
+
+
+
+
Input(1/5):
+
+
+
*
*
*
OTUB1
*
OTUB1
30
50
40
30
Supplementary Figure S3
