# Supplementary material for: OTUB1 inhibits the ubiquitination and degradation of FOXM1 in breast cancer and epirubicin resistance
Source: Oncogene. 2015 Jul 6;35(11):1433–44. doi: 10.1038/onc.2015.208 (PMC4606987; doi:10.1038/onc.2015.208)
Supplement: Supplementary Figure S4 [file onc2015208x6.ppt]

## Slide 1
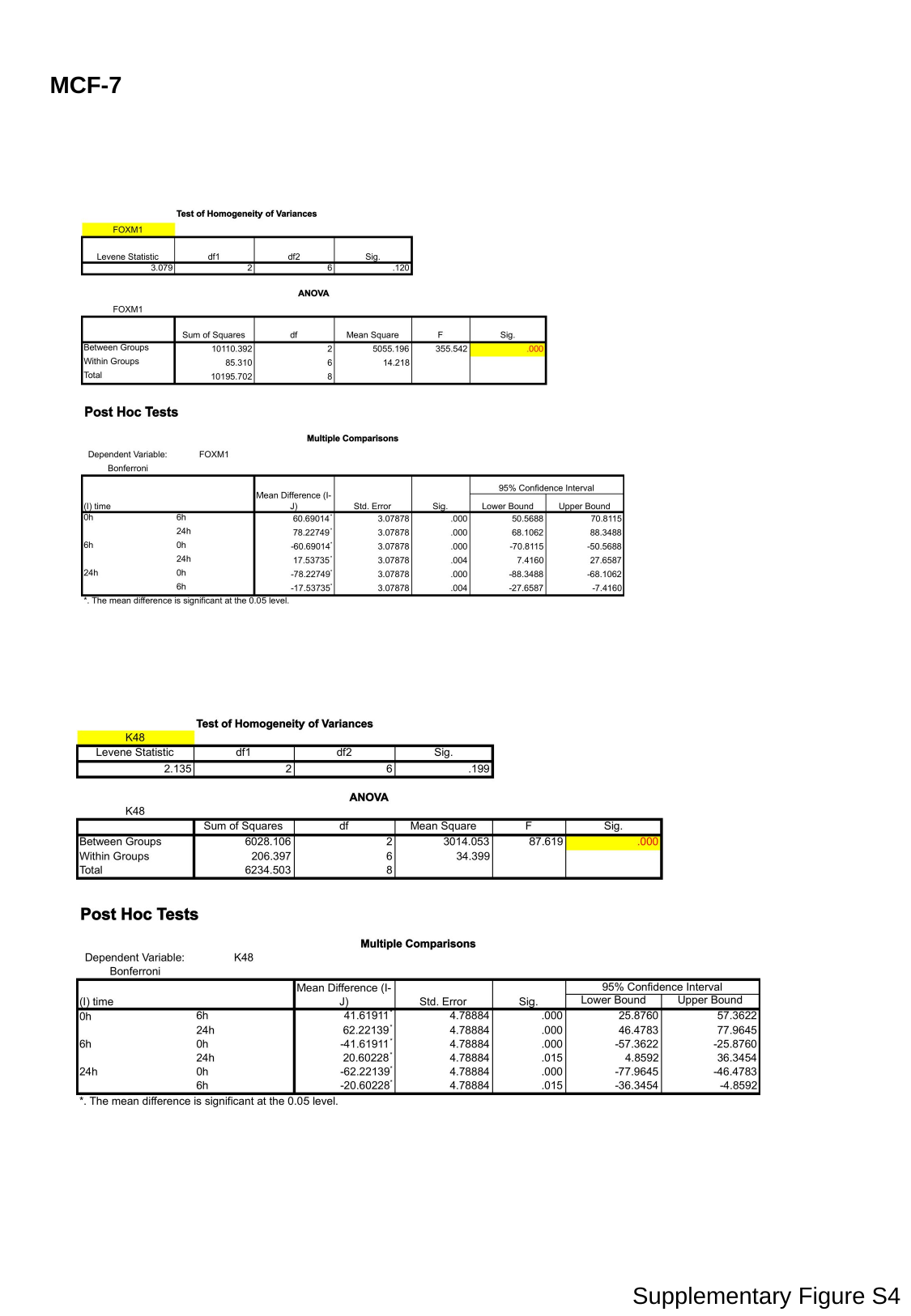

MCF-7
Supplementary Figure S4

## Slide 2
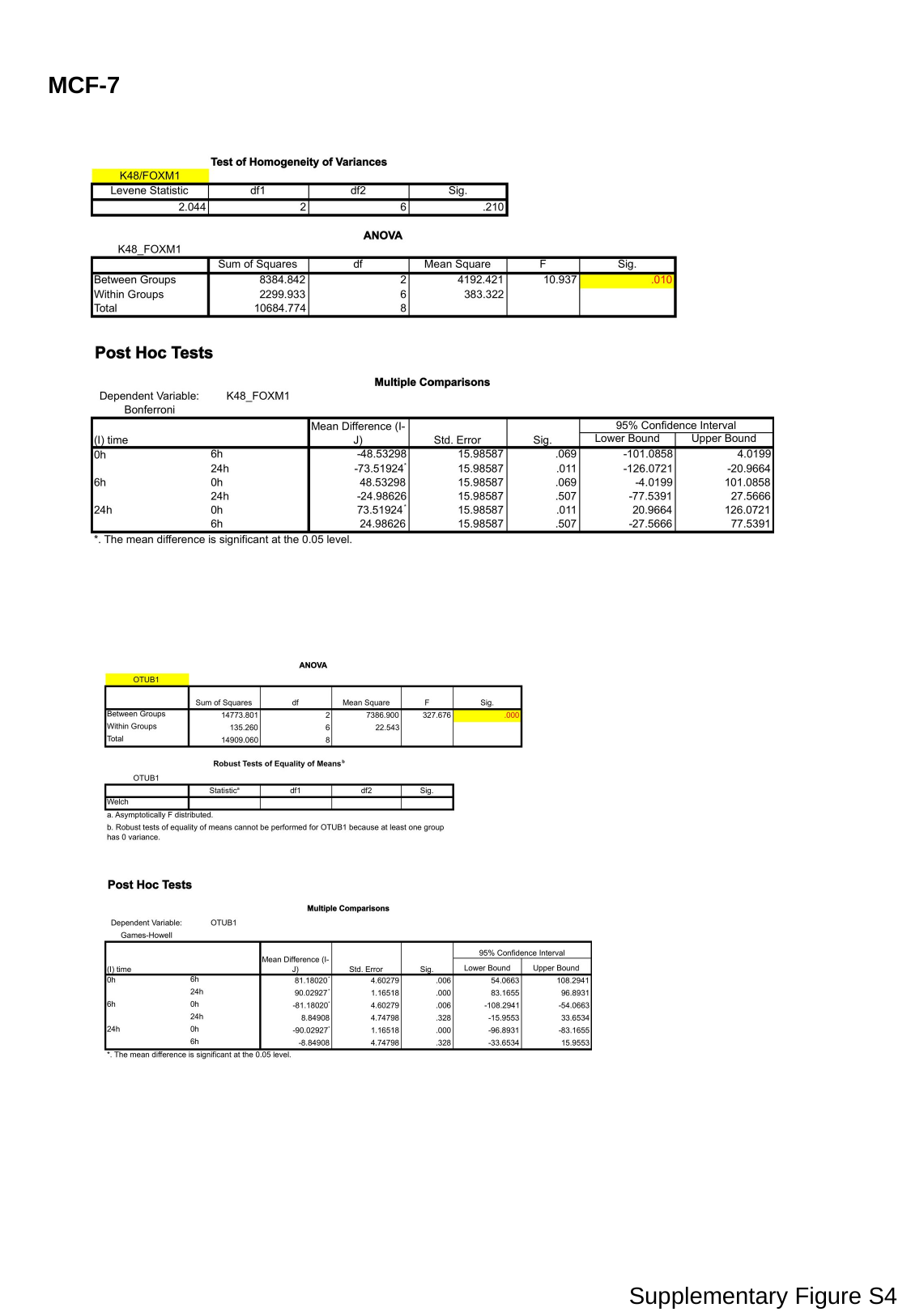

MCF-7
Supplementary Figure S4

## Slide 3
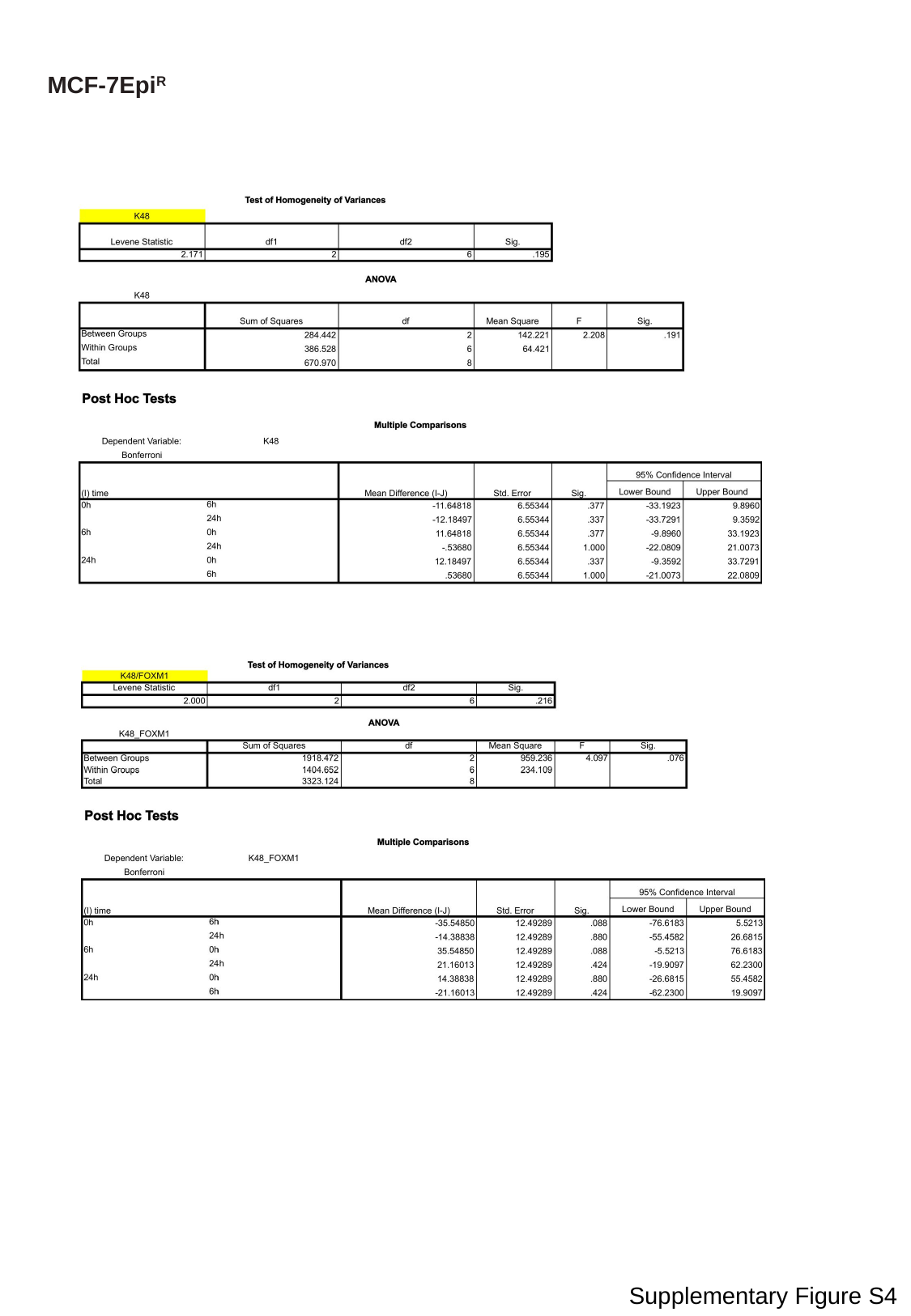

MCF-7EpiR
Supplementary Figure S4

## Slide 4
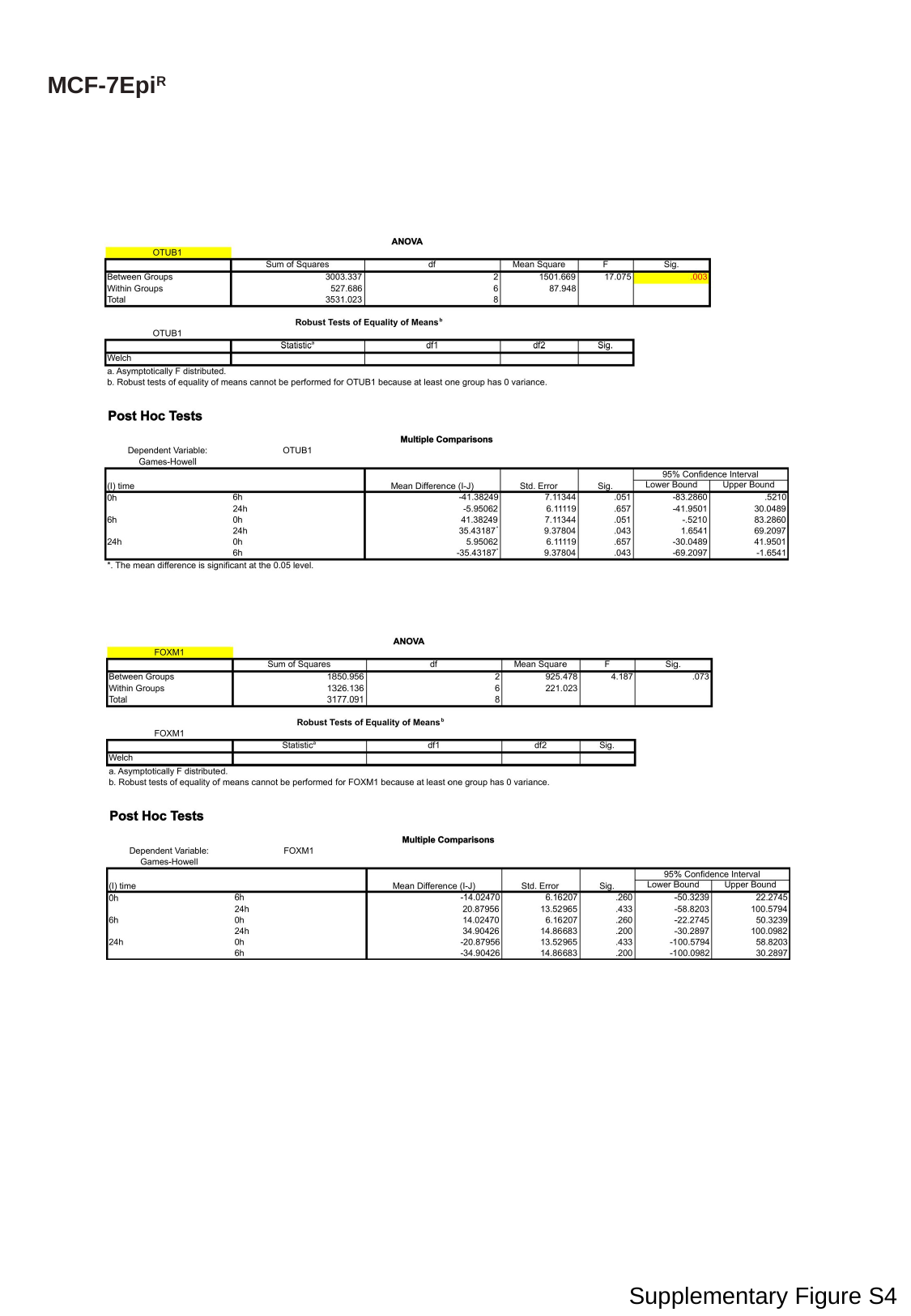

MCF-7EpiR
Supplementary Figure S4
