# Supplementary material for: OTUB1 inhibits the ubiquitination and degradation of FOXM1 in breast cancer and epirubicin resistance
Source: Oncogene. 2015 Jul 6;35(11):1433–44. doi: 10.1038/onc.2015.208 (PMC4606987; doi:10.1038/onc.2015.208)
Supplement: Supplementary Figure S5 [file onc2015208x7.ppt]

## Slide 1
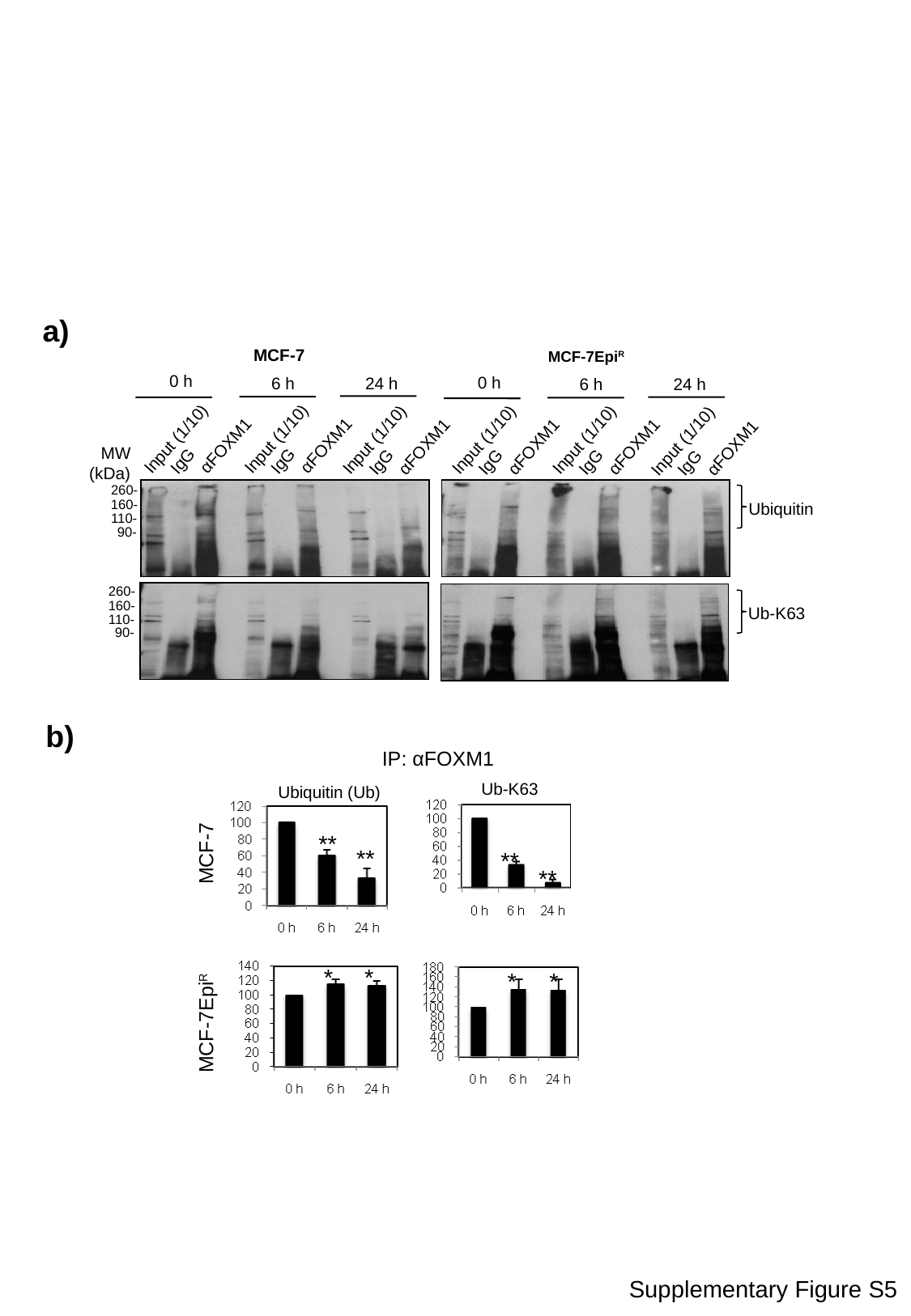

a)
MCF-7
MCF-7EpiR
0 h
0 h
6 h
24 h
6 h
24 h
Input (1/10)
Input (1/10)
Input (1/10)
Input (1/10)
Input (1/10)
Input (1/10)
αFOXM1
αFOXM1
αFOXM1
αFOXM1
αFOXM1
αFOXM1
MW
(kDa)
IgG
IgG
IgG
IgG
IgG
IgG
260-
160-
Ubiquitin
110-
90-
260-
160-
110-
90-
Ub-K63
b)
IP: αFOXM1
Ub-K63
Ubiquitin (Ub)
**
MCF-7
**
**
**
*
*
*
*
MCF-7EpiR
Supplementary Figure S5
