# Supplementary material for: OTUB1 inhibits the ubiquitination and degradation of FOXM1 in breast cancer and epirubicin resistance
Source: Oncogene. 2015 Jul 6;35(11):1433–44. doi: 10.1038/onc.2015.208 (PMC4606987; doi:10.1038/onc.2015.208)
Supplement: Supplementary Figure S6 [file onc2015208x8.ppt]

## Slide 1
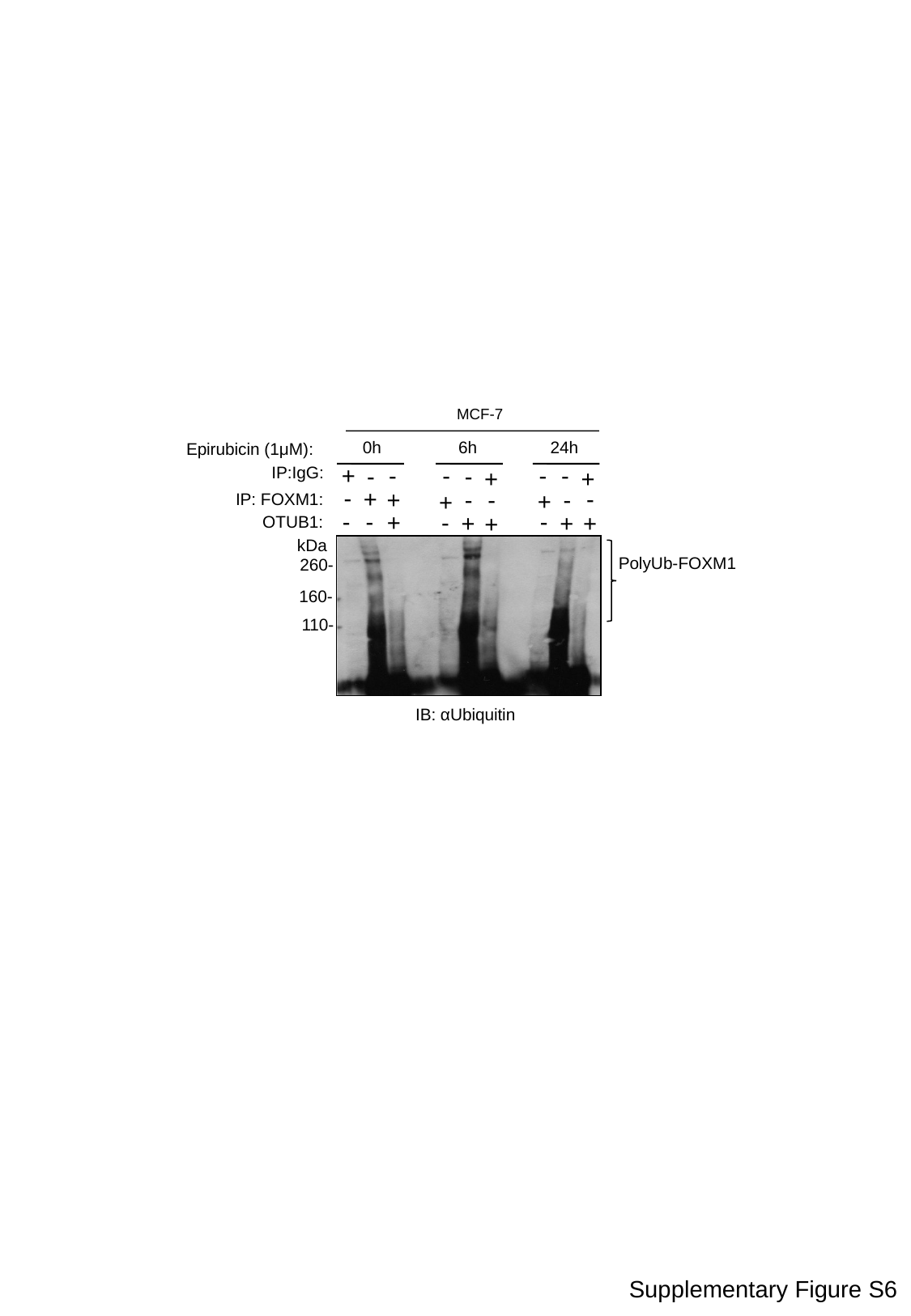

MCF-7
0h
6h
24h
Epirubicin (1μM):
-
-
+
-
-
-
-
IP:IgG:
+
+
-
+
+
-
-
-
-
+
+
IP: FOXM1:
-
-
-
+
-
+
+
+
+
OTUB1:
kDa
PolyUb-FOXM1
260-
160-
110-
IB: αUbiquitin
Supplementary Figure S6
