# Supplementary material for: OTUB1 inhibits the ubiquitination and degradation of FOXM1 in breast cancer and epirubicin resistance
Source: Oncogene. 2015 Jul 6;35(11):1433–44. doi: 10.1038/onc.2015.208 (PMC4606987; doi:10.1038/onc.2015.208)
Supplement: Supplementary Figure S7 [file onc2015208x9.ppt]

## Slide 1
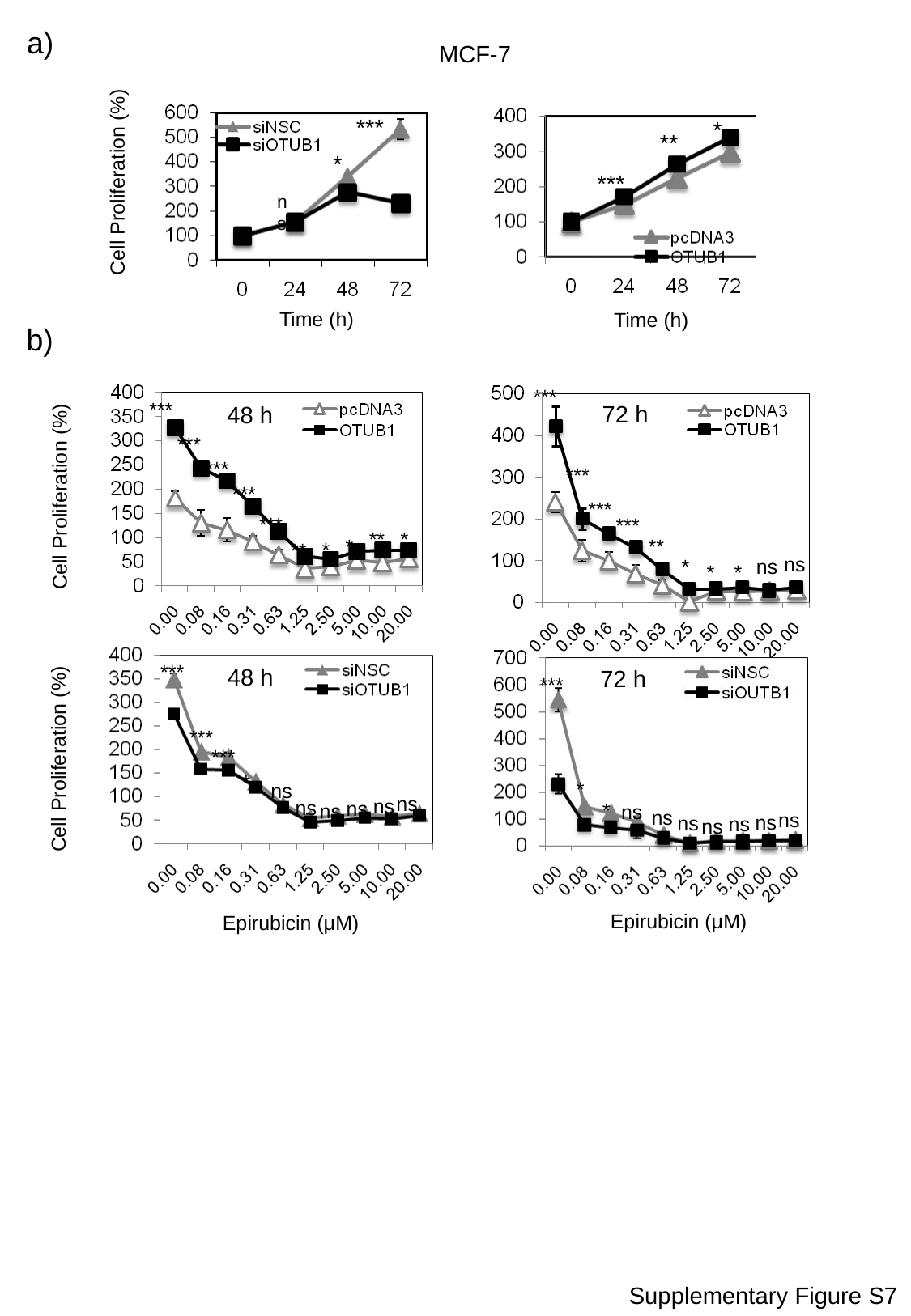

a)
MCF-7
***
*
**
*
***
Cell Proliferation (%)
ns
Time (h)
Time (h)
b)
***
***
***
***
***
*
**
*
*
**
***
***
***
***
**
ns
*
ns
*
*
72 h
48 h
Cell Proliferation (%)
***
48 h
72 h
***
***
***
Cell Proliferation (%)
*
*
ns
ns
ns
ns
ns
*
ns
ns
ns
ns
ns
ns
ns
ns
Epirubicin (μM)
Epirubicin (μM)
Supplementary Figure S7
