# Supplementary material for: OTUB1 inhibits the ubiquitination and degradation of FOXM1 in breast cancer and epirubicin resistance
Source: Oncogene. 2015 Jul 6;35(11):1433–44. doi: 10.1038/onc.2015.208 (PMC4606987; doi:10.1038/onc.2015.208)
Supplement: Supplementary Figure S8 [file onc2015208x10.ppt]

## Slide 1
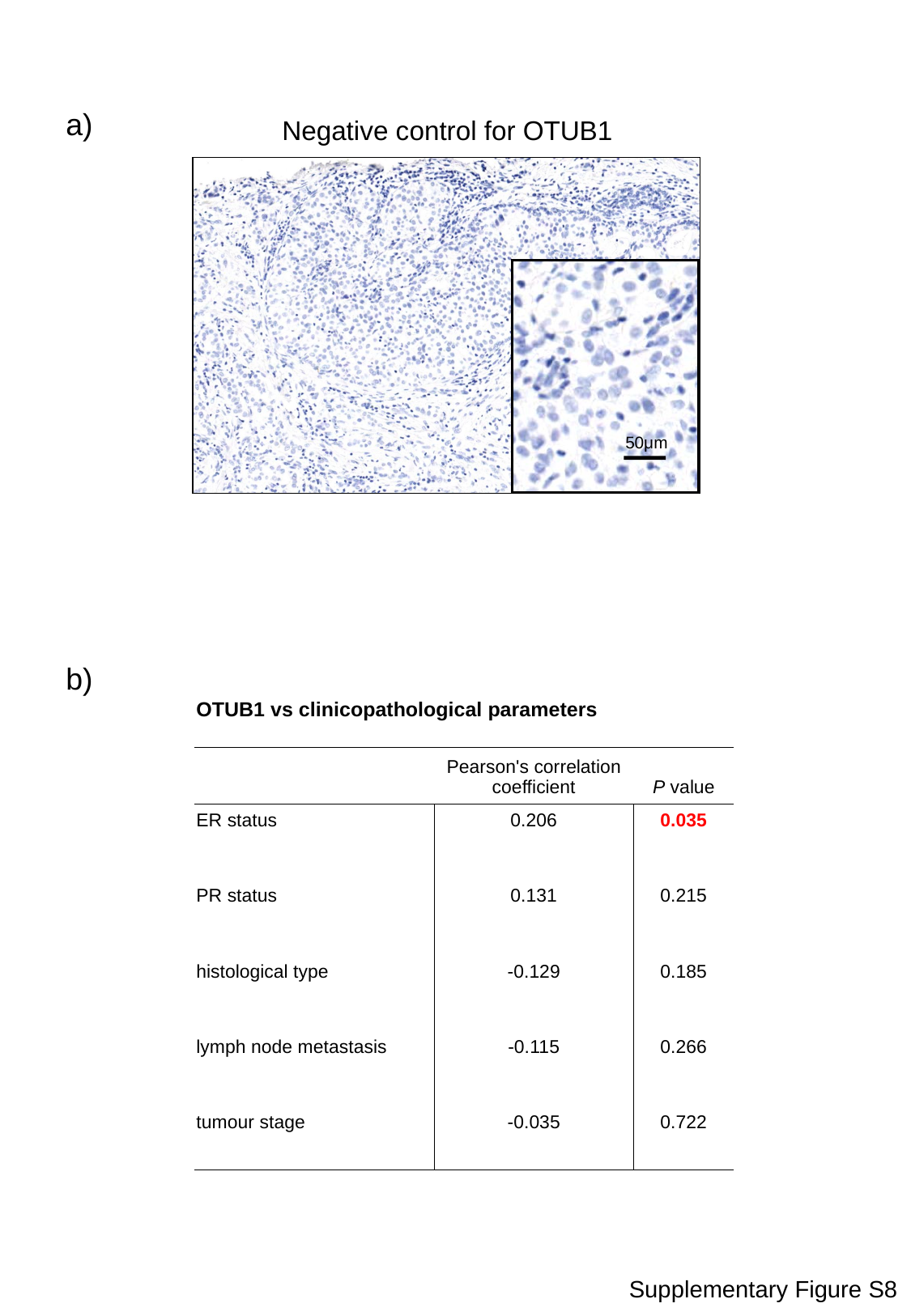

a)
# Negative control for OTUB1
50μm
b)
| OTUB1 vs clinicopathological parameters | | |
| --- | --- | --- |
| | Pearson's correlation coefficient | P value |
| ER status | 0.206 | 0.035 |
| | | |
| PR status | 0.131 | 0.215 |
| | | |
| histological type | -0.129 | 0.185 |
| | | |
| lymph node metastasis | -0.115 | 0.266 |
| | | |
| tumour stage | -0.035 | 0.722 |
| | | |
Supplementary Figure S8
