# Supplementary material for: OTUB1 inhibits the ubiquitination and degradation of FOXM1 in breast cancer and epirubicin resistance
Source: Oncogene. 2015 Jul 6;35(11):1433–44. doi: 10.1038/onc.2015.208 (PMC4606987; doi:10.1038/onc.2015.208)
Supplement: Supplementary Figure S9 [file onc2015208x11.ppt]

## Slide 1
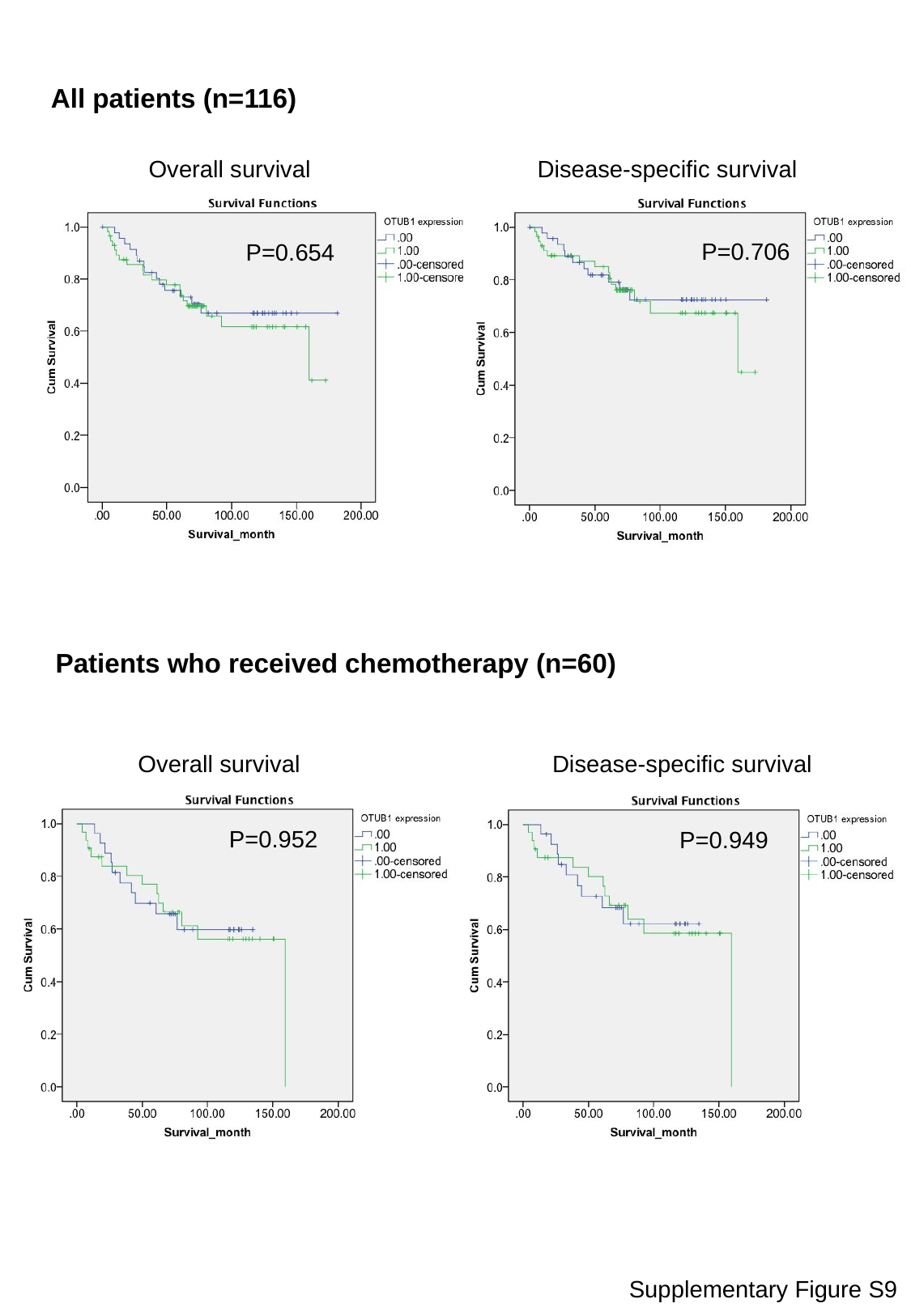

All patients (n=116)
Disease-specific survival
Overall survival
P=0.706
P=0.654
Patients who received chemotherapy (n=60)
Overall survival
Disease-specific survival
P=0.952
P=0.949
Supplementary Figure S9
