# Supplementary material for: OTUB1 inhibits the ubiquitination and degradation of FOXM1 in breast cancer and epirubicin resistance
Source: Oncogene. 2015 Jul 6;35(11):1433–44. doi: 10.1038/onc.2015.208 (PMC4606987; doi:10.1038/onc.2015.208)
Supplement: Supplementary Figure S10 [file onc2015208x12.ppt]

## Slide 1
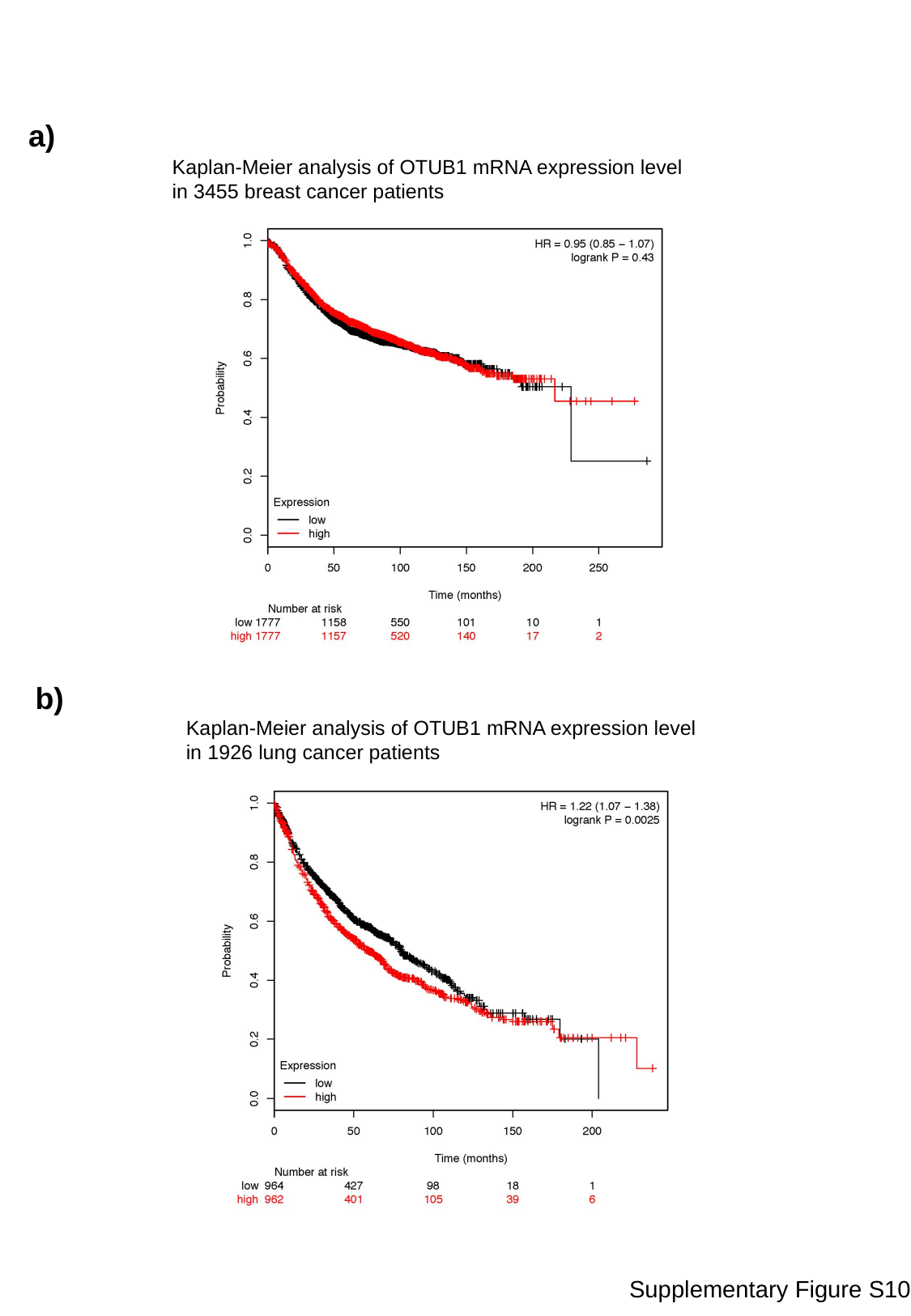

a)
Kaplan-Meier analysis of OTUB1 mRNA expression level
in 3455 breast cancer patients
b)
Kaplan-Meier analysis of OTUB1 mRNA expression level
in 1926 lung cancer patients
Supplementary Figure S10
