# Supplementary material for: OTUB1 inhibits the ubiquitination and degradation of FOXM1 in breast cancer and epirubicin resistance
Source: Oncogene. 2015 Jul 6;35(11):1433–44. doi: 10.1038/onc.2015.208 (PMC4606987; doi:10.1038/onc.2015.208)
Supplement: Supplementary Figure S11 [file onc2015208x13.ppt]

## Slide 1
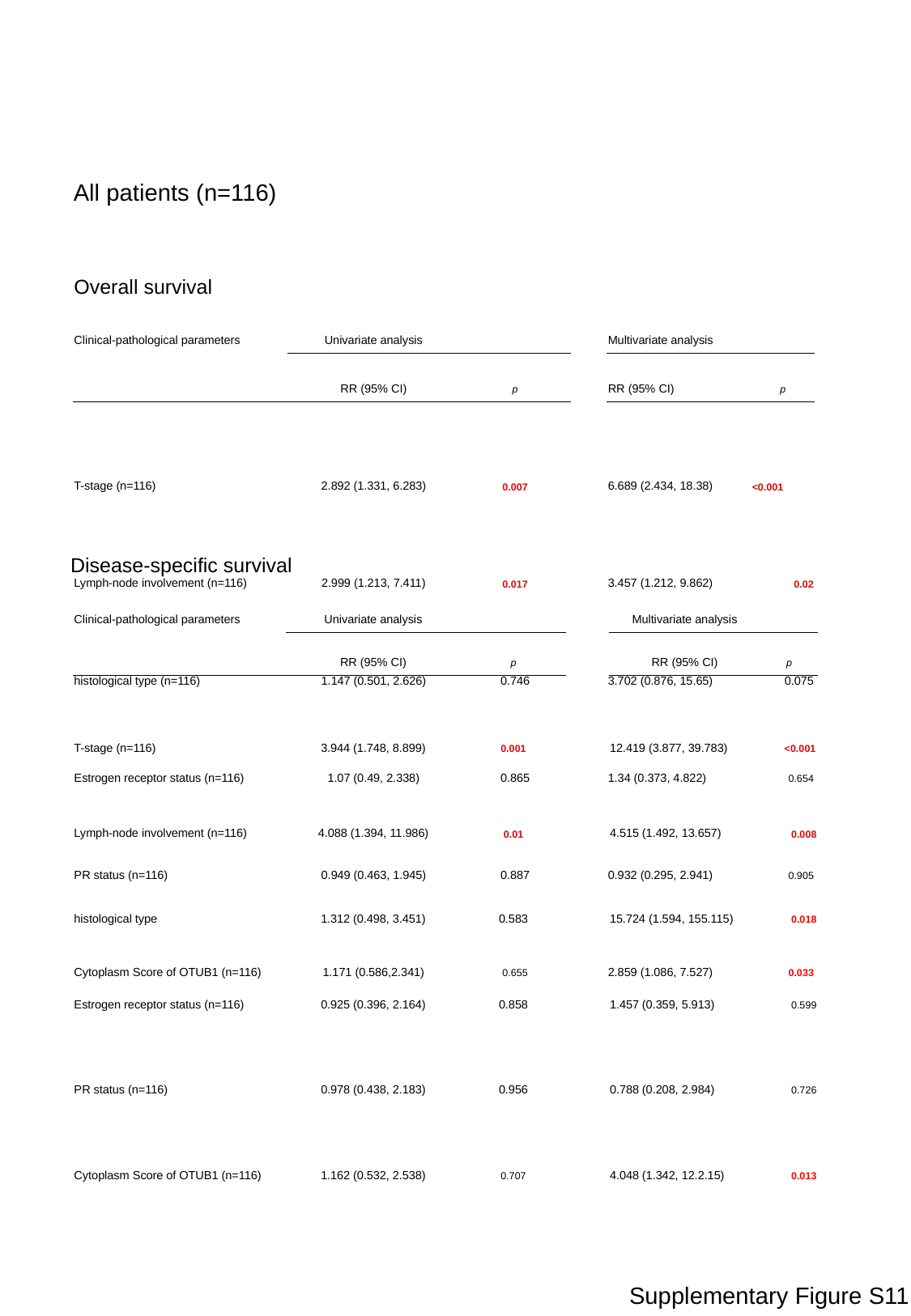

All patients (n=116)
Overall survival
| Clinical-pathological parameters | Univariate analysis | | | Multivariate analysis | |
| --- | --- | --- | --- | --- | --- |
| | RR (95% CI) | p | | RR (95% CI) | p |
| | | | | | |
| T-stage (n=116) | 2.892 (1.331, 6.283) | 0.007 | | 6.689 (2.434, 18.38) | <0.001 |
| | | | | | |
| Lymph-node involvement (n=116) | 2.999 (1.213, 7.411) | 0.017 | | 3.457 (1.212, 9.862) | 0.02 |
| | | | | | |
| histological type (n=116) | 1.147 (0.501, 2.626) | 0.746 | | 3.702 (0.876, 15.65) | 0.075 |
| | | | | | |
| Estrogen receptor status (n=116) | 1.07 (0.49, 2.338) | 0.865 | | 1.34 (0.373, 4.822) | 0.654 |
| | | | | | |
| PR status (n=116) | 0.949 (0.463, 1.945) | 0.887 | | 0.932 (0.295, 2.941) | 0.905 |
| | | | | | |
| Cytoplasm Score of OTUB1 (n=116) | 1.171 (0.586,2.341) | 0.655 | | 2.859 (1.086, 7.527) | 0.033 |
Disease-specific survival
| Clinical-pathological parameters | Univariate analysis | | | Multivariate analysis | |
| --- | --- | --- | --- | --- | --- |
| | RR (95% CI) | p | | RR (95% CI) | p |
| | | | | | |
| T-stage (n=116) | 3.944 (1.748, 8.899) | 0.001 | | 12.419 (3.877, 39.783) | <0.001 |
| | | | | | |
| Lymph-node involvement (n=116) | 4.088 (1.394, 11.986) | 0.01 | | 4.515 (1.492, 13.657) | 0.008 |
| | | | | | |
| histological type | 1.312 (0.498, 3.451) | 0.583 | | 15.724 (1.594, 155.115) | 0.018 |
| | | | | | |
| Estrogen receptor status (n=116) | 0.925 (0.396, 2.164) | 0.858 | | 1.457 (0.359, 5.913) | 0.599 |
| | | | | | |
| PR status (n=116) | 0.978 (0.438, 2.183) | 0.956 | | 0.788 (0.208, 2.984) | 0.726 |
| | | | | | |
| Cytoplasm Score of OTUB1 (n=116) | 1.162 (0.532, 2.538) | 0.707 | | 4.048 (1.342, 12.2.15) | 0.013 |
Supplementary Figure S11
