# Supplementary material for: OTUB1 inhibits the ubiquitination and degradation of FOXM1 in breast cancer and epirubicin resistance
Source: Oncogene. 2015 Jul 6;35(11):1433–44. doi: 10.1038/onc.2015.208 (PMC4606987; doi:10.1038/onc.2015.208)
Supplement: Supplementary Figure S12 [file onc2015208x14.ppt]

## Slide 1
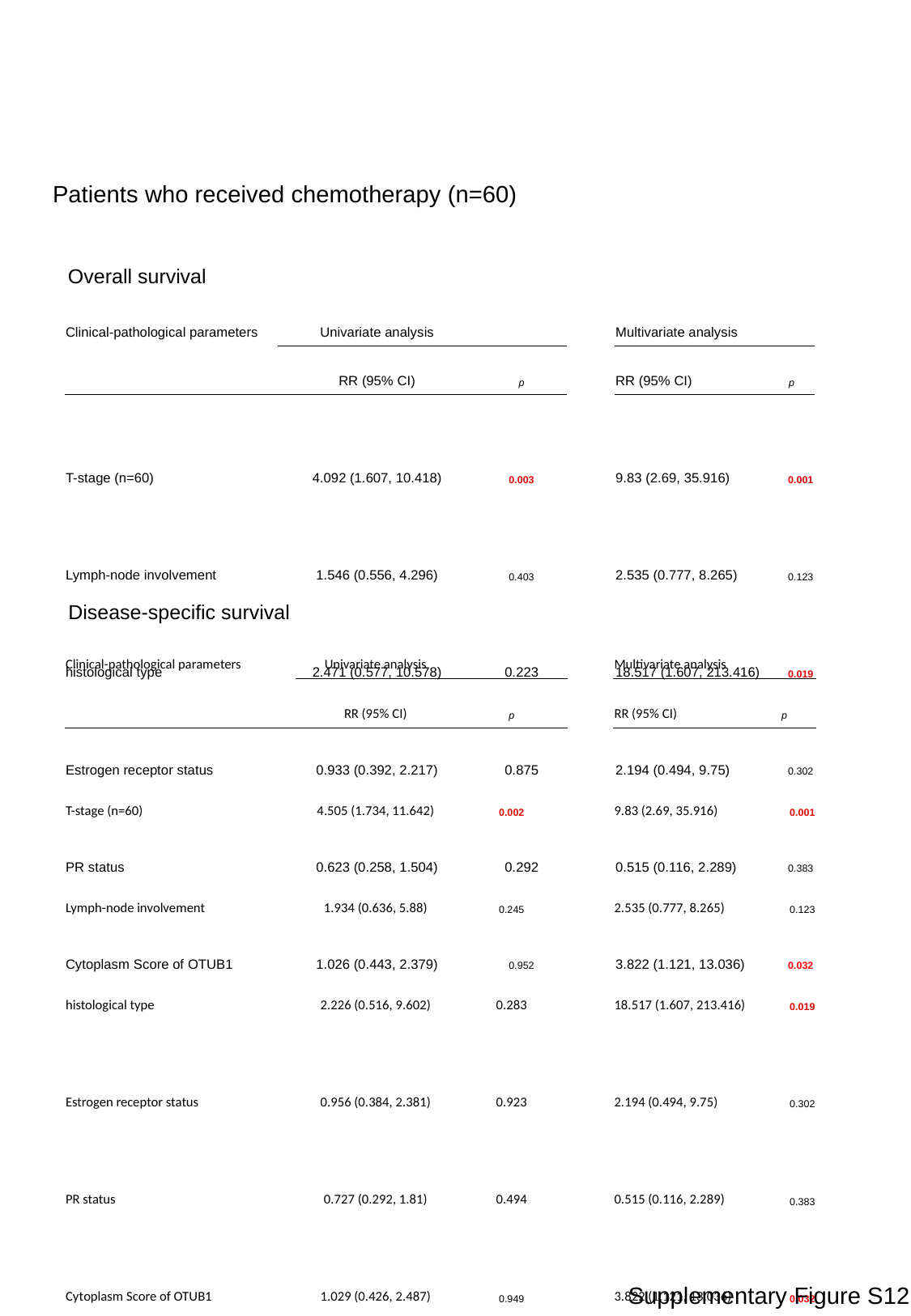

Patients who received chemotherapy (n=60)
Overall survival
| Clinical-pathological parameters | Univariate analysis | | | Multivariate analysis | |
| --- | --- | --- | --- | --- | --- |
| | RR (95% CI) | p | | RR (95% CI) | p |
| | | | | | |
| T-stage (n=60) | 4.092 (1.607, 10.418) | 0.003 | | 9.83 (2.69, 35.916) | 0.001 |
| | | | | | |
| Lymph-node involvement | 1.546 (0.556, 4.296) | 0.403 | | 2.535 (0.777, 8.265) | 0.123 |
| | | | | | |
| histological type | 2.471 (0.577, 10.578) | 0.223 | | 18.517 (1.607, 213.416) | 0.019 |
| | | | | | |
| Estrogen receptor status | 0.933 (0.392, 2.217) | 0.875 | | 2.194 (0.494, 9.75) | 0.302 |
| | | | | | |
| PR status | 0.623 (0.258, 1.504) | 0.292 | | 0.515 (0.116, 2.289) | 0.383 |
| | | | | | |
| Cytoplasm Score of OTUB1 | 1.026 (0.443, 2.379) | 0.952 | | 3.822 (1.121, 13.036) | 0.032 |
Disease-specific survival
| Clinical-pathological parameters | Univariate analysis | | | Multivariate analysis | |
| --- | --- | --- | --- | --- | --- |
| | RR (95% CI) | p | | RR (95% CI) | p |
| | | | | | |
| T-stage (n=60) | 4.505 (1.734, 11.642) | 0.002 | | 9.83 (2.69, 35.916) | 0.001 |
| | | | | | |
| Lymph-node involvement | 1.934 (0.636, 5.88) | 0.245 | | 2.535 (0.777, 8.265) | 0.123 |
| | | | | | |
| histological type | 2.226 (0.516, 9.602) | 0.283 | | 18.517 (1.607, 213.416) | 0.019 |
| | | | | | |
| Estrogen receptor status | 0.956 (0.384, 2.381) | 0.923 | | 2.194 (0.494, 9.75) | 0.302 |
| | | | | | |
| PR status | 0.727 (0.292, 1.81) | 0.494 | | 0.515 (0.116, 2.289) | 0.383 |
| | | | | | |
| Cytoplasm Score of OTUB1 | 1.029 (0.426, 2.487) | 0.949 | | 3.822 (1.121, 13.036) | 0.032 |
Supplementary Figure S12
